# Longitudinal outcome evaluations of Interdisciplinary Multimodal Pain Treatment programmes for patients with chronic primary musculoskeletal pain: A systematic review and meta‐analysis

**DOI:** 10.1002/ejp.1875

**Published:** 2021-11-05

**Authors:** Stefan Elbers, Harriët Wittink, Sophie Konings, Ulrike Kaiser, Jos Kleijnen, Jan Pool, Albère Köke, Rob Smeets

**Affiliations:** ^1^ Research group Lifestyle & Health Research Centre Healthy and Sustainable Living University of Applied Sciences Utrecht Utrecht The Netherlands; ^2^ Department of Rehabilitation Medicine Research School CAPHRI Faculty of Health, Life Sciences and Medicine Maastricht University Maastricht The Netherlands; ^3^ Department of Health Innovation and Technology Fontys University of Applied Sciences Eindhoven The Netherlands; ^4^ Comprehensive Pain Center Medical Faculty Technical University Dresden Dresden Germany; ^5^ University Hospital Carl Gustav Carus Dresden Dresden Germany; ^6^ Department of Family Medicine Research School CAPHRI Faculty of Health, Life Sciences and Medicine Maastricht University Maastricht The Netherlands; ^7^ Centre of Expertise in Pain and Rehabilitation Adelante, Maastricht The Netherlands; ^8^ South University of Applied Sciences Heerlen The Netherlands; ^9^ CIR Revalidatie Eindhoven The Netherlands

## Abstract

**Background and objectives:**

Although Interdisciplinary Multimodal Pain Treatment (IMPT) programmes share a biopsychosocial approach to increase the wellbeing of patients with chronic pain, substantial variation in content and duration have been reported. In addition, it is unclear to what extent any favourable health outcomes are maintained over time. Therefore, our first aim was to identify and analyse the change over time of patient‐related outcome measures in cohorts of patients who participated in IMPT programmes. Our second aim was to acquire insight into the heterogeneity of IMPT programmes.

**Databases and data treatment:**

The study protocol was registered in Prospero under CRD42018076093. We searched Medline, Embase, PsycInfo and Cinahl from inception to May 2020. All study selection, data extraction and risk of bias assessments were independently performed by two researchers. Study cohorts were eligible if they included adult patients with chronic primary musculoskeletal pain for at least 3 months. We assessed the change over time, by calculating pre‐post, post‐follow‐up and pre‐follow‐up contrasts for seven different patient‐reported outcome domains. To explore the variability between the IMPT programmes, we summarized the patient characteristics and treatment programmes using the intervention description and replication checklist.

**Results:**

The majority of the 72 included patient cohorts significantly improved during treatment. Importantly, this improvement was generally maintained at follow‐up. In line with our expectations and with previous studies, we observed substantial methodological and statistical heterogeneity.

**Conclusions:**

This study shows that participation in an IMPT programme is associated with considerable improvements in wellbeing that are generally maintained at follow‐up. The current study also found substantial heterogeneity in dose and treatment content, which suggests different viewpoints on how to optimally design an IMPT programme.

**Significance:**

The current study provides insight into the different existing approaches regarding the dose and content of IMPT programs. This analysis contributes to an increased understanding of the various approaches by which a biopsychosocial perspective on chronic pain can be translated to treatment programs. Furthermore, despite theoretical and empirical assertions regarding the difficulty to maintain newly learned health behaviors over time, the longitudinal analysis of health outcomes did not find a relapse pattern for patients who participated in IMPT programs

## 1 INTRODUCTION

Interdisciplinary multimodal pain treatment (IMPT) programmes are recognized as treatment of choice for patients with chronic pain (Gatchel et al., 2014; Turk, 2003). Since the 1970s these programmes have evolved towards interventions that combine (cognitive) behavioural approaches with exercise, medical treatment and education based on a biopsychosocial model. The aim of these programmes is not to target pain itself, but to help patients to optimize daily life functioning and to increase social, physical and psychological wellbeing (Gatzounis et al., 2012; Kaiser et al., 2017; Penney & Haro, 2019). This approach is typically provided by rehabilitation centres or hospitals and requires the expertise of an interdisciplinary team of healthcare providers. Generally, these disciplines cover the biopsychosocial spectrum and continuously coordinate their treatment activities and align them to patient‐specific goals.

Despite common historical roots and a biopsychosocial perspective on chronic pain, substantial variation in content, duration and outcome evaluations of IMPT programmes has been reported. For example, systematic reviews found that the total treatment duration varied between 6.4 and 196.8 h, programmes were delivered in both inpatient and outpatient settings, and pain‐related disability was measured with 12 different measurement instruments (Kamper et al., 2014; Scascighini et al., 2008; Waterschoot et al., 2014). This variability not only hinders a meaningful interpretation of pooled effect sizes but it also reflects uncertainty regarding optimal dose, content and the selection of measurement instruments (Waterschoot et al., 2014).

A second problem regarding the current evidence‐based IMPT programmes is that it is unclear to what extent treatment gains are maintained over time. Although RCTs often indicate a statistically significant effect compared to control interventions, post‐treatment assessments still suggest a considerable impact on daily life functioning (Kamper et al., 2014). From a clinical perspective, this indicates that most patients continue to experience the burdening effect of pain after treatment. This may be problematic, as the newly learned pain management strategies are considered to be fragile and vulnerable to disruptions (e.g. unexpected exacerbations of symptoms or an unforeseen event in the personal context or nocebo's). Continuing occurrences of pain interference could prompt pre‐treatment coping strategies, resulting in a declined effect over time (Carver & Scheier, 2017; Vlaeyen et al., 2016). Although this so‐called ‘triangular relapse pattern’—with an improvement from pre‐intervention to post‐intervention, followed by an unfavourable trend at follow‐up—has been observed in other healthcare domains, this topic has been neglected in the field of pain rehabilitation (Brouwer et al., 2019; Morley, 2008; Opozda et al., 2016; Turk & Rudy, 1991; Wilson, 2010; Wood & Neal, 2016). To understand the impact of these programmes on patients' ability to self‐regulate their wellbeing after completion of the treatment programme, it is crucial to assess the change of IMPT outcomes over time.

To acquire insight into both evidence‐gaps, our first aim was to identify and analyse the change over time of key outcome measures in patients with chronic pain who participated in IMPT programmes. Therefore, the first research question is: How does the physical, psychological and social wellbeing of patients with chronic musculoskeletal pain who participated in IMPT programmes change over time? Our second aim was to explore the heterogeneity of study, patient, intervention and outcome characteristics: To what extent do cohorts vary with respect to study, patient and treatment characteristics?

## 2 LITERATURE SEARCH METHODS

### 2.1 Protocol and registration

The study was reported in line with the PRISMA guidelines (Moher et al., 2009, 2015) and the study protocol has been registered in PROSPERO under CRD42018076093.

### 2.2 Search

We performed our search in Medline and Embase via OVID, and PsycInfo and Cinahl via EBSCOhost from inception to May 2020. The search string was developed by experienced reviewers (SE and JK) and consisted of multiple blocks that were combined with Boolean operators (see File S1). Each block included free‐text words as well as specific subject headings. In addition, we searched for grey literature including unpublished studies in the Dart Europe, Open access Theses and Dissertations, NDLTD, ClinicalTrials.gov and WHO ICTRP databases. For each included study, we also performed forward (in Google Scholar) and backward reference searches.

### 2.3 Eligibility criteria

Randomized controlled trials, as well as case series and cohort studies were included. Study cohorts had to include adult patients with chronic primary musculoskeletal pain for at least 3 months that was primarily perceived in musculoskeletal structures (e.g. bones, joints, muscles or related soft tissues; Treede et al., 2015, 2019). In case of mixed cohorts, at least 75% of the patients had to experience musculoskeletal pain. The criteria for IMPT programmes were based on the definition of Gatchel et al. and had to include (a) a common philosophy treatment in line with the biopsychosocial model of pain; (b) a treatment component where patients actively participated by means of tasks, training and/or exercise; (c) at least three different healthcare professionals from various disciplines that provided the interdisciplinary treatment; (d) a single facility where each patient received treatment (Gatchel et al., 2014). This last criterion excluded care‐network settings, but not multicenter trials. Although structured team meetings are considered an important aspect of IMPT programmes (Kaiser et al., 2017), we did not include this as an inclusion criterion, because we expected that not all studies would explicitly report this. Our outcomes were based on the criteria developed by the Initiative on Methods, Measurement, and Pain Assessment in Clinical Trials and included physical functioning, pain interference, depression, anxiety, emotional functioning, anger, self‐efficacy, social functioning and pain intensity (see protocol for rationale; Dworkin et al., 2005; Turk et al., 2003). The study had to include at least one outcome that was measured at two‐time points: prior to treatment and at least 12 months after the intervention was completed. Studies that focused on patients with post‐surgical pain or cancer pain, as well as studies that solely included patients on the basis of specific comorbidity (e.g. depression) were excluded. Articles published in other languages than English, German or Dutch were also excluded.

### 2.4 Study selection, data extraction and risk of bias

All study selection, data extraction and risk of bias assessments were independently performed by at minimum two different researchers (UK and SE for articles in German, SK, SE and MK for articles in other languages). Researchers used pre‐tested forms and compared their input to reach a consensus. In case of disagreement, the study was discussed with other researchers (HW and RS) for a final decision. Study selection was performed in two rounds. In the screening round, abstracts were screened using the Rayyan software package (Ouzzani et al., 2016). Subsequently, full‐text studies were assessed on all eligibility criteria.

From the extraction round onwards, we considered patient cohorts—not journal articles—as our primary unit of analysis. In case of multiple articles describing the same cohort, we combined these sources to construct a complete overview of the development over time. The first published article that met our eligibility criteria was used as the primary source and we consulted additional sources, such as protocols or follow‐up studies if they contained additional relevant information. If the information sources did not contain all data items of interest, we did not contact the study authors but coded this as ‘not reported’ in our dataset. Our data extraction form included all items from the template for intervention description and replication (TIDieR) checklist to describe the content of the treatment programme in detail (File S2; Hoffmann et al., 2014). Risk of Bias was assessed with the Joanna Briggs Institute Checklist for Case Series, which included 10 criteria (Moola et al., 2017). A response of ‘no’ to any one of the items resulted in a high risk of bias, unless we found a clear indication of a limited impact of that item on the overall study outcome. The risk of bias form, including the scoring instructions, are available in the online multimedia appendix.

### 2.5 Data analysis

The data extraction form included sample size (per measurement moment), age, sex, pain duration, nationality, method of recruitment, patient eligibility criteria, exclusion criteria, study design, type of outcome measures, and outcomes for all available time points on measurement instruments of interest. If treatment intensity was expressed in days, we assumed 6 h of treatment per day. Because IMPT programmes are generally considered as a treatment of last resort, we specifically paid attention to obtaining information on attrition (Jeffery et al., 2011). We obtained pre‐, post‐ and final‐follow‐up sample sizes to calculate attrition rates for post‐treatment and follow‐up. When a cohort presented data for two or more outcome measures within one domain, we selected the most commonly used instrument.

#### 2.5.1 Descriptive analysis

To investigate the heterogeneity between the included IMPT programmes, study, patient and intervention characteristics were summarized in tables. Intervention descriptions were extracted and each separate component was then classified into one of 10 possible categories. These components indicate the various means by which each IMPT programme aims to optimize daily life functioning and wellbeing. Education referred to modalities that were primarily concerned with the transfer of information from healthcare providers or experts to patients. All modalities regarding physical training, such as stretching, hydrotherapy and walking were categorized as exercise. Graded activity was only coded if the modality explicitly used the term graded activity or if the activities gradually and time‐contingent increased after a baseline measurement. Modalities that described (cognitive) behavioural approaches, including problem‐solving training, exposure in vivo, rational emotive therapy or ACT were classified as (cognitive) behavioural treatment. Breathing techniques, autogenic training, mindfulness, and applied relaxation techniques were classified as relaxation. Although self‐management, defined as ‘the intrinsically controlled ability of an active, responsible, informed and autonomous individual to live with the medical, role and emotional consequences of his chronic condition(s) in partnership with his social network and the healthcare provider(s)' (Van De Velde et al., 2019), is likely to be influenced various treatment components, some IMPT programmes included specific treatment sessions where coping with pain, setting realistic life goals and problem‐solving skills were discussed. These types of sessions were classified as generic self‐management skill training. Pharmacological treatment was only coded when medication was provided in response to chronic pain. Medication withdrawal procedures were coded as ‘other’. Workplace visits, and ergonomic advice at the workplace were coded as workplace advice. The category body awareness included physical awareness and psychomotor exercises that aimed to improve the recognition of bodily signals. The last category—team meetings—was only coded when the patient actively participated in the team meetings. The categories were inductively developed by first extracting and then clustering the modalities of the first search into global categories (by SE and SK). In the final dataset, these 10 categories covered more than 90% of the treatment modalities. All remaining modalities were coded as ‘other’. The description of each of the modalities and the classification were registered. A similar process was performed for healthcare providers. The following professions were coded as ‘physician’: occupational physician, rehabilitation physician, general practitioner and not otherwise specified physician. Other physician specialists (e.g. psychiatrist, orthopaedic surgeon, anesthesiologist) who were mainly involved in consulting instead of a coordinating role were coded as ‘other’. Disciplines such as clinical psychologists, general psychologists and behavioural therapists were classified as ‘psychologist’. Physical therapists and physiotherapists were classified as ‘physical therapist’. Social workers and social counsellors were classified as ‘social worker’. Occupational therapists and nurses were classified accordingly.

To assess to what extent treatment programmes aligned their programme with individual patient characteristics and preferences (i.e. tailoring), we classified each programme into low, medium or high tailoring. We defined low tailoring as any form of personalized goal‐setting, because this would allow patients to relate treatment content and progress to their personal situation. All studies received at minimum a ‘low’ tailoring classification because we assumed that all interdisciplinary programmes require some form of collaborative goal‐setting at the start of treatment. We classified programmes as medium tailoring, when they selected or optionally provided specific treatment components based on patient‐specific needs or preferences. High tailoring involved a fully personalized treatment programme, with varying duration and treatment activities and modules, based on each patient's clinical assessment.

#### 2.5.2 Main data analysis

In addition to pain intensity, we included seven key outcome measures as outcomes in this analysis, divided over three domains: physical health, mental health and social health. For physical health, we included physical functioning and pain interference. We extracted of the outcomes depression, anxiety, anger, and self‐efficacy beliefs within the mental health domain. For social health we only included social functioning. All outcomes were defined in the study protocol. For each of these outcomes that were present within a cohort, we used the available data to calculate effect sizes for pre‐post, post‐follow‐up and pre‐follow‐up contrasts. To calculate effect sizes, we used the method of Becker et al's standardized mean change (1988), with the modifications that were suggested by Morris (2000). The model assumes that the outcomes are normally distributed at both time points, with separate means but equal variances. Furthermore, the model corrects for a pre‐post within‐group correlation. Because we did not have access to the original data of the included cohorts, we imputed this value (Lipsey & Wilson, 2001). For all studies, we imputed the median correlation (*r* = .59) of a meta‐analysis that investigated the range of within‐group correlation values in active treatment groups (Balk et al., 2012). This value is comparable to other studies that have imputed within‐group meta‐analyses (Clond, 2016; Roberts et al., 2017). In addition, a sensitivity analysis for the within‐subject correlation is available in the multimedia appendix for all *r*‐values between 0 and 1. Sample sizes lower than *n* = 10 were not included in the analysis, because this could lead to inaccurate estimates of the standardized mean gain (Morris, 2000). In some interventions, the main treatment programme was followed by follow‐up treatment activities to enhance maintenance. In these situations, we considered end of treatment as the moment that the main treatment programme (ie. that covered the core of the treatment procedures) ended. Hence, follow‐up meetings, booster sessions or reinforcement sessions were not considered as main treatment and could continue after post‐treatment assessments. All assessments within 1 month after end of treatment were considered as a ‘post’ measure. We used the last available time point for the follow‐up contrast. We calculated standard deviations from standard errors by multiplying them with the square root of the corresponding sample size (Higgins et al., 2019). If medians and range were provided, we used the formula of Hozo et al. (2005) to estimate the mean value and corresponding SD. For studies that presented change scores, we calculated final value mean scores and imputed the baseline standard deviation. If the latter was not available, the study was not included in the meta‐analysis. For medians and interquartile ranges (IQRs), we estimated means and SDs using the assumption that the IQR width is 1.35 SD (Higgins et al., 2019). In case of missing measures of variability at follow‐up, we imputed the baseline value or otherwise used the mean SD of the remaining trials that reported on that outcome. If data of the cohort was presented for different subgroups, we calculated one composite mean and SD. For data that was only presented in figures (e.g. boxplots), we measured the central tendency and measure of dispersion if the figure was of sufficient quality.

Subsequently, we summarized the effect sizes per outcome, by describing the direction of effect for each of the included cohorts over time. We a priori decided not to perform any pooling, because this was not in line with our study aims and we expected substantial heterogeneity among the included studies. To facilitate interpretation of the effect sizes, we re‐expressed the median pre‐post effect size on the most commonly used measurement instrument, using the weighted standard deviation of all available post‐intervention scores of that instrument. To assess the statistical heterogeneity of the study outcomes we also calculated the *I* ^2^ and the *Q* test for each outcome domain at every time point. A statistically significant *Q* test rejects the hypothesis that all effect sizes are equal (Huedo‐Medina et al., 2006). In addition, the *I* ^2^ index provides an indication of the proportion of variability in observed effects that is either due to between‐study variability or due to within‐study variability (ie. sampling error; Borenstein et al., 2017). This analysis was performed with the *R* metaphor package in RStudio (R Core Team, 2013; RStudio Team, 2020; Viechtbauer, 2010).

#### 2.5.3 Exploratory data analysis

To further explore the included cohorts, we developed an online multimedia appendix that contains interactive forest plots for each outcome, time series and study characteristic tables. The appendix can be accessed via https://stefanelbers.shinyapps.io/deployment/. The time series show the development over time of a measurement on a standardized scale (expressed as a percentage of the maximum score of each particular measurement instrument), as well as the raw scores and standard deviations. To standardize the scores, we obtained the distance of each mean and the unfavourable end of the scale, divided by the total distance of the scale and multiplied by 100. These plots also allow for the comparison of specific cohorts over time with respect to a particular outcome. To accommodate future updates of this systematic review, the appendix also contains contact information to encourage readers to pinpoint any inaccuracies or to suggest cohorts that have not yet been included in the current review. Finally, all data extraction files are listed in this appendix, including any comments that have been made regarding handling specific difficulties with that specific cohort (e.g. dealing with change scores, or imputing missing SDs). File S3 contains the R code that has been used for all analyses in this review as well as the deployment of the appendix. These files can also be accessed in a Github repository via: https://github.com/stefanelbers/impt.meta_analysis/tree/master/deployment.

## 3 RESULTS

### 3.1 Results of the search

**FIGURE 1:**
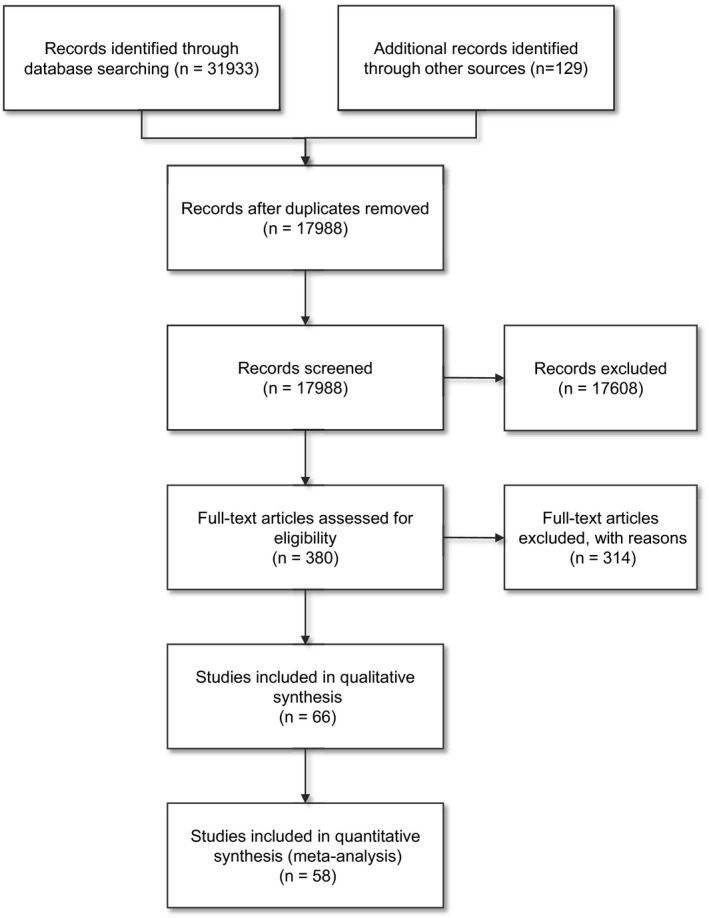
Flow chart of study records

The initial search was performed in May 2019 and was updated in May 2020 using the last date of the original search as the beginning date for the update. In total, the search yielded 31,933 hits. After deduplication, 17,988 studies remained. The screening of title and abstracts yielded 380 hits. In the final selection round, we obtained the full‐text versions and included 66 studies. 314 studies were excluded: 50 studies due to study design or publication type, 41 studies related to patient criteria, 89 studies due to intervention criteria, 89 studies because they did not include outcomes within the scope of this study or did not include a follow‐up measurement of 12 months or longer, 38 studies were duplicates and seven studies due to language or inclusion of patients with specific comorbidities. Seven of the included studies did not provide the necessary data (ie. central tendency and measure of dispersion for each time point) to be included the quantitative analyses and were only included in the characteristics tables. The study flow is depicted in Figure 1.

### 3.2 Characteristics of the included studies

#### 3.2.1 Study characteristics

We included 37 case series (i.e. longitudinal studies with prospectively collected data on one cohort of patients), 20 RCTs, four N‐RCTs and five other types of study design. In total, these studies provided data on 76 cohorts with a median final follow‐up measurement of 12 months (range 12–120). Pre‐treatment sample sizes of the cohorts ranged from 10 to 2089, with a median of 97. After filtering out cohorts that reported no dropout in the study flow (assuming a complete case analysis) the median dropout ratio for the 55 remaining cohorts was 8.56 (range 0–42.11) at post‐treatment and 16.67 (range = −3.85 to 62.17) at the final follow‐up, using the posttreatment sample size as a reference. Six of the 66 studies were evaluated as low risk of bias, indicating that in the majority of the cohorts we identified at least one factor that threatened the internal validity. Statistical analysis and attrition (84.8%), unclear inclusion criteria (43.9%), incomplete inclusion of participants (60.6%), and incomplete reporting of clinical information of the participants (34.8%) were the most frequent reasons for assigning an unclear or high risk of bias. File S3 provides an overview of the risk of bias assessment.

#### 3.2.2 Patient characteristics

The majority of the included patients were treated in European or North‐American countries (94.6%). The mean distribution of sex was 68.7% female (SD = 18.1%) The average mean age of the cohorts was 44.353 years (SD = 4.49). The median pain duration prior to treatment, reported in 40 of the included cohorts, was 76.8 months (range = 16–217.2). Generally, patients were referred to the programme by their primary care physician or medical specialist. Table 1 provides a summary of the patient characteristics. The multimedia appendix includes a more extensive and searchable table.

**TABLE 1:** Patient characteristics

Author (year) Cohort	Cohort name	Study sample size	Dropout (%)	Lost to follow‐up (5)	Nationality	% females	Mean age (SD)	Patient group	Mean pain duration (months)
Abbasi et al. (2012): a	Spouse‐assisted multidisciplinary pain management program	36	10	0	Iran	88	45 (10)	Low back pain	107.5
Abbasi et al. (2012): b	Patient‐oriented multidisciplinary pain management program	36	0	16.67	Iran	88	45 (10)	Low back pain	107.5
Beaudreuil et al. (2010)	Functional restoration program	39	0	0	France	28	42.9 (8.2)	Low back pain	28.6
Bendix et al. (1998)a	Functional restoration (A)	238	14.55	6.38	Denmark	67.4	42 (NA)	Chronic low back pain	NA
Bendix et al. (1998): b	Functional restoration (B)	238	10.87	9.76	Denmark	70.27	41 (NA)	Chronic low back pain	NA
Bendix et al. (2000)	Functional Restoration	138	13.56	5.88	Denmark	66.1	42.1 (NA)	Chronic low back pain	NA
Bergström et al. (2001)	Vocational rehabilitation program	156	NA	NA	Sweden	48	42.5 (9.5)	neck pain or back pain	38
Bergström et al. (2014)	Multimodal rehabilitation	43	9.3	12.82	Sweden	80	41 (NA)	Disabling chronic pain	NA
Bileviciute‐Ljungar and Norrefalk (2014): a	Rehabilitation cohort (1998)	88	NA	NA	Sweden	84	43.2 (2.1)	Persistent musculoskeletal pain	NA
Bileviciute‐Ljungar and Norrefalk (2014): b	Rehabilitation (2003)	88	NA	NA	Sweden	89.47	39.5 (1.9)	Persistent musculoskeletal pain	NA
Borys et al. (2015)	Multimodal therapy	155	0	59.35	Germany	55.6	58.3 (10.4)	Chronic low back pain	217.2
Brendbekken et al. (2016)	Multidisciplinary intervention	284	2.84	37.96	Norway	53.9	41.3 (NA)	Musculoskeletal pain	NA
Cardosa et al. (2012)	MENANG program	102	0	0	Malaysia	64.3	42.87 (9.87)	Chronic pain	66.81
de Rooij et al. (2014)	Multidisciplinary treatment programme	138	9.77	5	Netherlands	95	45.04 (10.3)	Chronic widespread pain	84
Dysvik et al. (2013)	Multidisciplinary pain management programme	117	11.11	8.65	Norway	79	45 (11.25)	Chronic non‐malignant pain	89.9
Frost et al. (2000)	Functional restoration	129	1.63	19.83	United Kingdom	45	43 (9)	Constant pain	NA
Gantschnig et al. (2017)	BAI‐REHA	30	13.33	−3.85	Swiss	43	44.83 (12.57)	ICD−10 diagnosis of chronic musculoskeletal pain	NA
Gerdle et al. (2016)	Multimodal rehabilitation program	464	NA	NA	Sweden	81.6	38.1 (10.1)	Chronic pain	83.8
Grahn et al. (2000)	Multidisciplinary rehabilitation	236	NA	NA	Sweden	82	44.3 (9.1)	Prolonged musculoskeletal disease as main diagnosis	68.4
Gustafsson et al. (2002)	Multimodal, multidisiciplinary outpatient rehabilitation programme	23	4.35	22.73	Sweden	100	43.8 (10.7)	Fibromyalgia or widespread chronic pain	158.4
Hafenbrack et al. (2013): a	Berlin	681	2.86	55.51	Germany	49.2	45.81 (9.22)	Back pain	NA
Hafenbrack et al. (2013): b	Hamburg	681	3.18	55.26	Germany	55.3	44.95 (9.84)	Back Pain	NA
Haiduk et al. (2017)	Zurzacher Interdisziplinäres HWS Konzept (ZIHKo)	115	NA	NA	Switzerland	83.1	40.3 (12.3)	Chronic neck pain	17.2
Hållstam et al. (2016)	Multimodal rehabilitation	42	NA	NA	Sweden	90.5	43.6 (15.7)	Complex pain problems	NA
Hazard et al. (1989)	Functional restoration	64	1.69	31.03	USA	36	37 (4.3)	Low back pain	NA
Hildebrandt et al. (1996)	GRIP	98	1.1	6.67	Germany	48.9	41.7 (8.7)	Lumbal and unspecific back pain	NA
Huffman et al. (2019)	ICPRP	2089	19.53	62.17	USA	65.3	46.6 (13.6)	chronic non cancer pain	NA
Ibrahim et al. (2019)	Geneva MBR program	201	20.4	33.12	Switzerland	40.8	39.98 (10.06)	non‐specific LBP	NA
Jensen et al. (1997): a	multimodal cognitive behavioral treatment only (regular intervention)	63	6.67	10.71	Sweden	100	43 (9)	Nonspecific spinal pain without neurological signs	51
Jensen et al. (1997): b	Multimodal cognitive behavioral treatment (experimental program)	63	6.06	6.45	Sweden	100	45 (8)	Nonspecific spinal pain without neurological signs	44
Kääpä et al. (2006)	Multidisciplinary rehabilitation program	64	7.81	16.95	Finland	100	46 (7.9)	Low back pain	16
Koopman et al. (2004)	Multidisciplinary occupational training program	68	5.88	20.31	Netherlands	47.1	41.3 (8.91)	Low back pain	72
Lemstra and Olszynski (2005)	Multidisciplinary rehabilitation	43	16.28	2.78	Canada	86	49.7 (9.57)	Chronic widespread pain	121.7
Letzel et al. (2019)	Multidisciplinary biopsychosocial rehabilitation	113	28.32	20.99	Germany	67.9	59.7 (10.7)	Chronic neck pain	NA
Mangels et al. (2009): a	Behavioral‐medical rehabilitation	363	0	6.19	Germany	78.8	49.5 (9)	Musculoskeletal pain	NA
Mangels et al. (2009): b	Behavioral‐medical rehabilitation+booster	363	0	6.72	Germany	75.6	48.3 (15.8)	Musculoskeletal pain	NA
Martín et al. (2012)	PSYMEPHY	180	10	13.89	Spain	93.46	49.07 (8.92)	Fibromyalgia	174.22
McAllister et al. (2005)	Multidisciplinary chronic pain program	276	20.65	54.79	United States	66.3	44.7 (9.7)	Chronic non‐malignant pain	NA
Meng et al. (2011)	New back school	382	11.17	16	Germarny	65.2	50.2 (7.6)	Chronic low back pain	NA
Merrick and Sjölund (2009)	Interdisciplinary rehabilitation	255	1.79	−1.21	Sweden	79	39 (NA)	Disabling chronic pain	NA
Merrick et al. (2012)	Multimodal rehabilitation	296	NA	NA	Sweden	76	39.2 (9.7)	Chronic pain	62.4
Monticone et al. (2013)	Multidisciplinary intervention	90	0	0	Italy	60	48.96 (7.97)	Chronic non‐specific low back pain	22.15
Monticone et al. (2016)	Multidisciplinary cognitive behavioural rehabilitation programme	150	1.33	12.16	Italy	62.67	53.2 (11.1)	Non‐specific low back pain	21.7
Nagel and Korb (2009)	Multimodal therapy	351	38.96	0	Germany	59.3	44.7 (NA)	Chronic nonspecific back pain	140.4
Nicholas et al. (2014): a	Interoceptive Exposure	140	10.61	28.81	Australia	51	42.05 (12.33)	Chronic pain	67.16
Nicholas et al. (2014): b	Distraction	140	8.11	41.18	Australia	55	43.22 (11.08)	Chronic pain	77.71
Olason (2004)	Interdisciplinary pain management program	158	24.05	NA	Iceland	70.9	39.5 (NA)	Chronic pain	NA
Oslund et al. (2009)	Comprehensive outpatient pain management program	108	0	42.5	USA	70.4	55 (11.47)	Patients with various chronic pain problems not specified	110.44
Persson et al. (2012)	Musculoskeletal interdisciplinary pain rehabilitation program	813	13.53	27.6	Sweden	79	40 (9.6)	Musculoskeletal pain	49.2
Pietilä‐Holmner et al. (2020)	Musculoskeletal interdisciplinary pain rehabilitation program	467	NA	NA	Sweden	85.5	43.6 (10.8)	Musculoskeletal chronic pain	NA
Reck et al. (2017)	Interdisciplinary multimodal pain therapy	71	NA	NA	Switzerland	54	43.59 (11.84)	Chronic non‐specific low back pain	NA
Richardson et al. (1994)	Pain management course	109	9.17	−10.1	United Kingdom	68	45 (10)	Chronic pain	128
Roche‐Leboucher et al. (2011)	Functional restoration	132	NA	NA	France	32.4	40.8 (7.4)	Non‐specific low back pain	NA
Semrau et al. (2015)	PASTOR	554	5	27.44	Germany	54.1	48.9 (8)	Low back pain	NA
Silvemark et al. (2014)	Pain rehabilitation programme	164	13.64	48.87	Sweden	69.5	37.4 (9.07)	Long‐term non‐malignant pain	68.4
Smeets et al. (2008): a	Graded activity with problem solving training	223	5.17	5.45	Netherlands	58.6	42.52 (9.67)	Non‐specific low‐back pain	68.33
Smeets et al. (2008): b	Combination treatment	223	9.84	3.64	Netherlands	37.7	40.67 (10.14)	Non‐specific low‐back pain	56.13
Spinhoven et al. (2004)	Merged cohorts: operant‐behavioral treatment +coping or group discussion (OPCO/OPDI)	148	11.97	12.62	Netherlands	63.5	39.8 (9.1)	Low back pain	117.6
Stein and Miclescu (2013)	Multidisciplinary rehabilitation	59	0	13.56	Sweden	86	48 (7.8)	Chronic non‐cancer pain	NA
Steinmetz et al. (2019)	Multimodal therapy	276	9.78	56.22	Germany	57.4	53.4 (10.6)	chronic spinal pain	
Strobel et al. (1998)	Interdisciplinary group treatment	32	NA	NA	Germany	87.5	49 (7.5)	Fibromyalgia (ACR criteria)	92.4
Tavafian et al. (2011)	Group‐based multidisciplinary rehabilitation program	197	4.12	25.81	Iran	73.2	44.6 (10.2)	Chronic low back pain	75.9
Thieme et al. (2003)	Operant pain therapy	61	4.76	0	Germany	100	46.61 (8.67)	Fibromyalgia	204.6
Van der Maas et al. (2015): a	Pain rehabilitation	114	42.11	21.21	Netherlands	71.1	45.4 (11.1)	Chronic musculoskeletal pain	NA
Van der Maas et al. (2015): b	Rehabilitation treatment +body awareness	114	8.16	48.89	Netherlands	91.8	38.6 (11.1)	Chronic musculoskeletal pain	NA
van Hooff et al. (2010)	Pain management programme	107	23.36	0	Netherlands	57	44.1 (8.4)	Low back pain	147.6
van Wilgen et al. (2009)	Inpatient multidisciplinary CBT programme	32	18.75	0	Netherlands	73	42 (11)	Chronic pain	96
Vendrig et al. (2000)	Multidisciplinary behavioural program for chronic pain	147	NA	NA	Netherlands	31	41.6 (8.5)	Chronic low back pain	46.3
Verkerk et al. (2011)	Functional recovery	1760	3.64	43.1	Netherlands	74.26	40.1 (10.6)	Chronic non‐specific low back pain	92.4
Volker et al. (2017)	Outpatient multidisciplinary programme	165	6.67	22.08	Netherlands	87	44.1 (12.9)	Chronic musculoskeletal pain	NA
Vowles et al. (2011)	ACT	187	8.56	36.84	United Kingdom	64.2	47.3 (11.4)	Chronic pain	96
Wagner et al. (2011)	Interdisciplinary treatment program	142	0	0	Canada	63.64	49.16 (10.03)	Chronic pain	32.99
Westman et al. (2006)	Early multimodal rehabilitation	91	NA	NA	Sweden	70	41.5 (NA)	Musculoskeletal pain and disability	NA
Williams et al. (1996): a	Inpatient pain management	323	28.57	3.33	United Kingdom	53	48.7 (11.6)	Chronic pain	NA
Williams et al. (1996): b	Outpatient pain management	323	7.32	18.42	United Kingdom	49	50.4 (11.7)	Chronic pain	NA
Zhuk et al. (2018)	Multimodal pain treatment	59	NA	85.68	Germany	62.7	45.27 (10.34)	Low back pain	NA

#### 3.2.3 Intervention characteristics

The treatment aims of the included programmes often involved multiple objectives, such as increasing physical activity, return to work, or the acquisition of pain self‐management skills. The median time span of the treatment duration was 5 weeks (range 1–15), with a mean intensity of 95.91 h (SD = 52.72). Twenty‐two cohorts (29.73%) included, at least partly, inpatient treatment programmes and two cohorts (2.67%) solely provided treatments to individuals, whereas the other cohorts at least partially provided treatment to groups. The majority of interdisciplinary treatment was provided in secondary or tertiary care settings, such as hospitals or rehabilitation centres, with a community centre (one cohort), hotel (one cohort) and a primary care setting (three cohorts) as exceptions. Exercise (93%), education (89%), relaxation (80%), generic self‐management skill training (74%), and (cognitive) behavioural treatment (70%) were included in the majority of the treatment programmes, whereas body awareness (25%), graded activity (16%), workplace advice (16%), pharmacological treatment (15%), and team meetings that included the patient (11%) were less frequently reported. The median number of these specific treatment modalities was 5 (range 2–8) per cohort. Many cohorts also included modalities that were categorized as ‘other’, such as assertiveness training, spinal mobilisations, group discussions, and assistance with withdrawal from pain medication. The median number of involved healthcare professionals was 4 (range 3–7), with physical therapists (97%), psychologists (93%), physicians (85%), occupational therapists (53%), nurses (34%) and social workers (32%) as respective frequencies. Other involved healthcare providers (described in 57% of the cohorts) included nutritionists, massage therapists and Qigong instructors.

Follow‐up treatment sessions were described in 22 (41%) of the cohorts and mainly consisted of group refresher meetings or follow‐up phone calls. Four cohorts included an extensive follow‐up module where parts of the treatment programme were continued for a prolonged period (Monticone et al., 2013, 2016; Tavafian et al., 2011; Westman et al., 2006). In total, 81% of the included studies provided low tailoring, 11% medium tailoring and 8% high tailoring. Table 2 depicts a general overview of the intervention characteristics, but the full table is displayed in the multimedia appendix.

**TABLE 2:** Intervention characteristics

Cohort	Treatment modalities	Healthcare providers						In/outpatient setting	Type of contact	Group size	Location	Time span (wks)	Duration (h)	Level of tailoring	Follow‐up sessions
		phy	psy	pt	ot	nur	swo								
Abbasi et al. (2012): a	ed, bt, re, sm, oth	1	1	1	0	0	0	Out	Mixed	6	Pain clinic	7	15	Low	No
Abbasi et al. (2012): b	ed, bt, re, sm, oth	1	1	1	0	0	0	Out	Mixed	6	Pain clinic	7	16	Low	No
Beaudreuil et al. (2010)	ex, re, oth	1	0	1	1	0	1	Out	Group	2–6	Hospital	5	138	Low	No
Bendix (1998): a	ed, ex, bt, re, oth	1	1	1	1	0	1	Out	Group	6–8	Pain clinic	3	135	Low	No
Bendix et al. (1998): b	ed, ex, bt, re, oth	1	1	1	1	0	1	Out	Group	6–8	Pain clinic	3	135	Low	No
Bendix et al. (2000)	ed, ex, re, sm, oth	1	1	1	1	0	1	Out	NA	6–8	Pain clinic	3	135	Low	No
Bergström et al. (2001)	ed, ex, re, ba, oth	0	0	1	0	0	0	In	Group	14	Rehab center	4	160	Medium	No
Bergström et al. (2014)	ed, ex, re, sm, ba, te, oth	1	1	1	1	0	1	Out	Group	10–12	Hospital	5	NA	Low	No
Bileviciute‐Ljungar and Norrefalk (2014): a	ed, ex, sm, wo, oth	1	1	1	1	1	1	Out	Mixed	8	Hospital	8	143	Low	No
Bileviciute‐Ljungar and Norrefalk (2014): b	ed, ex, sm, wo, oth	1	1	1	1	1	1	Out	Mixed	8	Hospital	8	143	Low	No
Borys et al. (2015)	ed, ex, bt, ph, ba, te	1	1	1	0	0	1	In	Mixed	NA	Hospital	3	168	Low	No
Brendbekken et al. (2016)	ex, sm, te	1	0	1	0	0	1	Out	Individual	NA	Hospital	NA	8	Low	yes
Cardosa et al. (2012)	ed, ex, bt, re, sm, oth	1	1	1	0	1	0	In	Group	NA	Hospital	2	60	Low	No
de Rooij et al. (2014)	ed, ex, bt, re, sm, oth	1	1	1	1	0	1	Out	Mixed	NA	Rehab center	7	49	High	Yes
Dysvik et al. (2013)	ed, ex, sm, ba, oth	1	1	1	0	1	0	Out	Group	8–12	Hospital	8	45	Low	Yes
Frost et al. (2000)	ed, ex, re, oth	1	1	1	0	0	0	Out	Group	max 5	NA	3	98	Low	Yes
Gantschnig et al. (2017)	ed, ex, bt, re, sm, ba, wo, oth	1	1	1	1	1	1	Mix	Mixed	NA	Hospital	12	108	Medium	No
Gerdle, Molander, Stenberg, Stalnacke, et al. (2016)	ed, bt, sm, oth	1	1	1	1	0	1	Out	Group	6–9	Hospital	7	140	Medium	No
Grahn et al. (2000)	ed, ex, bt, re, sm, ba, wo, oth	1	0	1	1	1	0	In	Mixed	NA	Rehab center	4	120	High	Yes
Gustafsson et al. (2002)	ed, ex, re, ba, oth	1	1	0	0	1	1	Out	Mixed	7–8	Hospital	12	96	Low	Yes
Hafenbrack et al. (2013): a	ed, ex, re, sm, ph, wo	1	1	1	0	0	0	Out	Group	NA	Pain clinic	4	120	Low	No
Hafenbrack et al. (2013): b	ed, ex, re, sm, ph, wo	1	1	1	0	0	0	Out	Group	NA	Pain clinic	4	120	Low	No
Haiduk et al. (2017)	ex, bt, re, sm	1	1	1	1	0	0	In	Mixed	NA	Hospital	4	108	Low	No
Hållstam et al. (2016)	ex, bt, re, sm, oth	1	1	1	0	1	0	Out	Mixed	NA	Hospital	13	65	Low	No
Hazard et al. (1989)	ed, ex, bt, re, sm, oth	0	1	1	1	0	0	Out	Mixed	NA	Rehab center	3	53	Low	No
Hildebrandt et al. (1996)	ed, ex, bt, re, sm, wo	1	1	1	0	1	1	Out	Group	8–10	Pain clinic	11	207	Medium	No
Huffman et al. (2019)	ed, ex, bt, oth	0	1	1	1	1	0	Out	Mixed	NA	Rehab center	3.5	166	Low	Yes
Ibrahim et al. (2019)	ed, ex, bt, re, sm, oth	1	1	1	1	0	0	Out	Group	4–6	Hospital	4	100	Low	Yes
Jensen et al. (1997): a	ed, ex, bt, re, sm, oth	1	1	1	0	1	0	In	Group	NA	Pain clinic	5	NA	Low	Yes
Jensen et al. (1997): b	ed, ex, bt, re, sm, oth	1	1	1	0	1	0	In	Group	NA	Pain clinic	5	NA	Low	Yes
Kääpä et al. (2006)	ed, ex, bt, re, ph, wo	1	1	1	1	0	0	Out	Mixed	6–8	Rehab center	8	70	Low	No
Koopman et al. (2004)	ex, ga, bt, re, oth	1	1	1	0	0	0	Out	Mixed	6–10	Rehab center	12	216	Low	No
Lemstra and Olszynski (2005)	ed, ex, re, sm, oth	1	1	1	0	0	0	Out	Mixed	NA	Community center	6	33	Low	No
Letzel et al. (2019)	ed, ex, bt, re, sm, oth	1	1	1	1	0	0	Out	Group	5–10	Hospital	3	44	Low	No
Mangels et al. (2009): a	ed, ex, re, sm, ph	0	1	1	1	0	0	In	Mixed	10–12	Hospital	NA	167	Low	No
Mangels et al. (2009): b	ed, ex, re, sm, ph	0	1	1	1	0	0	In	Mixed	10–12	Hospital	NA	167	Low	Yes
Martín et al. (2012)	ed, ex, bt, re, sm, ph	1	1	1	0	0	0	Out	Group	12	Hospital	6	21	Low	No
McAllister et al. (2005)	ex, re, sm, oth	1	0	1	0	1	0	Mix	Group	NA	NA	4	80	Low	Yes
Meng et al. (2011)	ed, ex, sm	0	1	1	0	0	0	In	Group	7–15	Hospital	3	163	Low	No
Merrick and Sjölund (2009)	ed, ex, bt, re, sm, ba, oth	1	1	1	1	0	1	Out	Mixed	NA	Hospital	5	67	Low	No
Merrick et al. (2012)	ed, ex, bt, re, sm, ba, te, oth	1	1	1	1	0	1	Out	Group	6–8	Hospital	4	65	Medium	No
Monticone et al. (2013)	ed, ex, bt, sm, oth	1	1	1	0	0	0	Out	Individual	NA	Rehab center	5	15	Low	Yes
Monticone et al. (2016)	ed, ex, bt, re, sm, oth	1	1	1	0	0	0	Out	Group	5	Rehab center	5	15	Low	Yes
Nagel and Korb (2009)	ed, ex, bt, re	1	1	1	0	1	0	Out	Group	8–9	Pain clinic	3.5	89	Low	No
Nicholas et al. (2014): a	ed, ex, bt, sm, oth	1	1	1	0	1	0	Out	Group	8–10	Hospital	3	115	Low	No
Nicholas et al. (2014): b	ed, ex, bt, re, sm	1	1	1	0	1	0	Out	Group	8–10	Hospital	3	115	Low	No
Olason (2004)	ed, ex, bt, re, sm, ph, ba, te, oth	1	1	1	1	1	1	In	Mixed	NA	Rehab center	7	NA	Medium	No
Oslund et al. (2009)	ed, ex, bt, re, sm	1	1	1	1	0	0	Out	Mixed	NA	University	4	120	Low	No
Persson et al. (2012)	ed, ex, bt, re, sm, ba, wo, te, oth	1	1	1	1	1	1	Out	Group	9	Hospital	5	126	Low	Yes
Pietilä‐Holmner et al. (2020)	ed, ex, bt, re, sm	1	1	1	1	0	1	Out	Mixed	NA	Primary care centers	8	20	Low	No
Reck et al. (2017)	ed, ex, bt, re, sm, wo	1	1	1	1	0	0	Out	Group	NA	Pain clinic	1	45	Low	Yes
Richardson et al. (1994)	ed, ex, ga, bt, re, sm, oth	0	1	1	1	1	0	Mix	Group	5	Hospital	NA	120	Low	No
Roche‐Leboucher et al. (2011)	ex, re, wo, te, oth	1	1	0	1	0	0	Out	Group	6–8	Rehab center	5	150	Low	No
Semrau et al. (2015)	ed, ex, re, sm, oth	1	1	1	0	0	1	In	Mixed	NA	Rehab center	3	48	Low	No
Silvemark et al. (2014)	ed, ex, bt, re, sm, ba	1	1	1	1	1	1	Out	Group	6–9	Hospital	5	175	Low	No
Smeets et al. (2008): a	ed, ga, bt, oth	1	1	1	1	0	1	Out	Group	Max 4	Rehab center	10	24	Low	No
Smeets et al. (2008): b	ed, ex, ga, bt, oth	1	1	1	1	0	1	Out	Group	Max 4	Rehab center	10	77	Low	No
Spinhoven et al. (2004)	ed, ga, bt, sm, oth	0	1	1	1	0	0	Mix	Mixed	NA	Rehab center	8	150	Low	No
Stein and Miclescu (2013)	ed, ex, bt, re, sm, ba, te, oth	1	1	1	1	0	0	Out	Group	6–8	Primary care setting	6	90	Low	No
Steinmetz et al. (2019)	ex, re, ph, oth	1	1	1	0	0	0	In	NA	NA	Hospital	2	15	High	No
Strobel et al. (1998)	ed, ex, ga, re, sm, ba, oth	1	1	1	0	1	0	In	Group	6	Rehab center	5	150	Low	No
Tavafian et al. (2011)	ed, ex, bt, re, sm, ph, oth	0	1	1	0	0	0	Out	Group	NA	University	1	9	Low	Yes
Thieme et al. (2003)	ed, ex, bt, sm	1	1	1	0	1	0	In	Group	5–7	Hospital	5	75	Low	No
Van der Maas et al. (2015): a	ed, ex, ga, bt, re, oth	1	1	1	1	0	0	Out	Group	4–6	Rehab center	12	94	Low	Yes
Van der Maas et al. (2015): b	ed, ex, ga, bt, re, ba, oth	1	1	1	1	0	0	Out	Group	4–6	Rehab center	12	109	Low	Yes
van Hooff et al. (2010)	ed, ex, bt, re, sm	0	1	1	1	0	0	In	Group	NA	Hotel facility	2	100	Low	No
van Wilgen et al. (2009)	ed, ex, ga, bt, re, sm, oth	1	1	1	0	0	0	Mix	Mixed	NA	Hospital	7	NA	High	No
Vendrig et al. (2000)	ed, ex, ga, ph, wo	0	1	1	0	0	0	Out	Group	NA	Rehab center	4	NA	Low	No
Verkerk et al. (2011)	ed, ex, re, sm, ba	1	1	1	0	0	0	Out	Group	6	Rehab center	9	48	Low	Yes
Volker et al. (2017)	ed, ex, bt, re	1	1	1	1	0	1	Out	Mixed	NA	Rehab center	15	NA	Low	No
Vowles et al. (2011)	ed, ex, bt, re, sm, ba	1	1	1	1	1	0	In	Group	NA	Hospital	3.5	114	Low	No
Wagner et al. (2011)	ed, ex, bt, re, sm	1	1	1	1	1	0	Out	Mixed	NA	Pain clinic	6	123	High	No
Westman et al. (2006)	ed, ex, bt, re, ba, oth	1	1	1	0	0	0	Out	Group	8–10	Primary care setting	8	140	Low	Yes
Williams et al. (1996): a	ed, ex, ga, bt, re, pm, oth	0	1	1	1	1	0	In	Group	10	Hospital	4	108	Low	No
Williams et al. (1996): b	ed, ex, ga, bt, re, pm, oth	0	1	1	1	1	0	Out	Group	10	Hospital	8	28	Low	No
Zhuk et al. (2018)	ed, ex, bt, re, sm, ba, oth	1	1	1	0	1	0	Out	Group	NA	Pain clinic	3	NA	Low	No

Abbreviations: ba, body awareness therapy; bt, (cognitive) behavioral therapy; ed, education; ex, exercise; ga, graded activity; nur, nurse; ot, occupational therapist; oth, other type of treatment modality; ph, pharmacological therapy; phy, physician; psy, psychologist; pt, physiotherapist; re, relaxation; sm, self‐management skills; swo, social worker; te, team meetings; wo, workplace advice.

#### 3.2.4 Effect of time

For all outcomes, the median pre‐post effect sizes show a favourable trend, indicating a positive change in health from pre to post‐treatment (range = 0.38–1.94). The post to final‐follow‐up effect sizes vary from −0.49 to 0.15, indicating different trends. The median effect sizes from pre to final follow‐up show an overall favourable change in health outcomes during the course of the study (range = 0.32 to 0.85). Table 3 shows the median effect size, range and the amount of statistical heterogeneity per contrast. The table also includes an overview of the number of cohorts that follow a particular pattern of effect over time, symbolized by different plotlines. For example, a statistically significant favourable pre‐post effect that is followed by no effect from post to follow‐up is represented by a positive slope, that flattens halfway.

**TABLE 3:** Distribution of effect sizes of pre‐post, post‐follow up and pre‐follow‐up contrasts for each outcome domain

Outcome	Pre‐post			Post‐final follow‐up			Pre‐post‐final follow‐up		Pre‐final follow‐up			*N* ^a^	Direction of effect^c^
	*N* ^a^	Mdn ES (range)	*Q* test/*I* ^2^	*N* ^a^	Mdn ES (range)	*Q* test/*I* ^2^	*N* ^a^	Pattern over time^b^	*N* ^a^	Mdn ES (range)	*Q* test/*I* ^2^		
Pain interference	36	0.72 (−0.21 to 4.93) RMDQ^2^: 3.21	*Q* (*df* = 35) = 1463.38*** *I* ^2^ = 99%	36	0.01 (−0.59 to 3.01)	*Q* (*df* = 35) = 4561.80*** *I* ^2^ = 95%	7	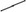	53	0.53 (−0.40 to 11.46)	*Q* (*df* = 52) = 1300.71*** *I* ^2^ = 99%	42	+
							22	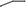					
												10	+/−
							4	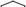					
												1	−
							3	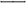					
Physical function	13	0.50 (0.04–3.26) FFbH^2^: 8.99	*Q* (*df* = 12) = 225.56*** *I* ^2^ = 99%	13	0.00 (−0.23 to 0.45)	*Q* (*df* = 12) = 32.64** *I* ^2^ = 68%	2	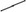	20	0.43 (0.08–3.59)	*Q* (*df* = 19) = 290.58*** *I* ^2^ = 98%	16	+
							8	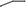					
												4	+/−
							3	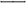					
Depression	31	0.71 (0.10–3.19) BDI^2^: 5.57	*Q* (*df* = 30) = 889.89*** *I* ^2^ = 95%	31	−0.17 (−1.29 to 0.33)	*Q* (*df* = 30) = 202.99*** *I* ^2^ = 88%	1	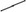	41	0.42 (−0.03 to 1.29)	*Q* (*df* = 40) = 467.63*** *I* ^2^ = 91%	31	+
							15	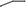					
							12	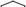				10	+/−
							2	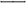					
							1	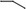					
Anxiety	12	0.45 (0.11–3.77) HADS‐A^2^: 1.94	*Q* (*df* = 11) = 293.37*** *I* ^2^ = 99%	12	−0.04 (−1.61 to 0.73)	*Q* (*df* = 11) = 66.07*** *I* ^2^ =97%	6	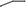	19	0.32 (0.02–1.18)	*Q* (*df* = 18) = 349.62*** *I* ^2^ = 92%	14	+
							4	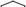					
							1	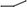				5	+/−
							1	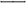					
Emotional functioning	6	0.88 (0.48–2.99) SF36 MH^2^: 10.63	*Q* (*df* = 5) = 111.42*** *I* ^2^ = 98%	6	−0.2 (−0.84 to 0.66)	*Q* (*df* = 5) = 55.96*** *I* ^2^ = 93%	2	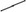	11	0.62 (−0.15 to 3.48)	*Q* (*df* = 10) = 202.74*** *I* ^2^ = 99%	10	+
							1	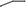				1	+/−
							3	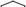					
Anger	1	1.944 SCL‐90h^2^: 3.88	NA	1	−0.49^d^	NA	1	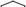	1	0.49^d^	NA	1	+
Self‐efficacy	9	0.88 (0.10–1.21) PSEQ^2^: 10.54	*Q* (*df* = 8) = 83.21*** *I* ^2^ = 87.38%	9	0.06 (−0.21 to 0.25)	*Q* (*df* = 8) = 7.51 *I* ^2^ = 0%	7	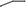	11	0.85 (0.16–1.32)	*Q* (*df* = 10) = 83.5*** *I* ^2^ =84%	10	+
							2	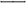				1	+/−
Social functioning	5	0.38 (−0.08 to 2.37) SF36srf^2^: 6.39	*Q* (*df* = 4) = 126.9*** *I* ^2^ = 98%	5	0.15 (−0.27 to 0.42)	*Q* (*df* = 4) = 17.6** *I* ^2^ = 77%	1	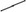	8	0.71 (0.02–2.39)	*Q* (*df* = 7) = 129.47*** *I* ^2^ = 97%	6	+
							1	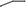					
							1	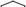					
							1	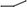				2	+/−
							1	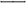					
Pain intensity	38	0.63 (−0.08 to 4.39) VAS^2^: 14.29	*Q* (*df* = 37) = 853.42*** *I* ^2^ = 99%	38	0.04 (−1.39 to 1.09)	*Q* (*df* = 37) = 761.98*** *I* ^2^ = 96%	10	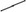	55	0.45 (−0.31 to 4.96)	*Q* (*df* = 54) = 1481.91*** *I* ^2^ = 98%	35	+
							14	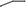					
												17	+/−
							6	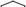					
							5	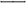				3	−
							3	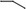					

Abbreviations: BDI, Beck Depression Inventory; *df*, degrees of freedom; ES, effect size; FFbH, Funktionsfragebogen Hannover; HADS‐A, Hospital Anxiety and Depression Scale, subscale Anxiety; Mdn, median; PSEQ, Pain Self‐efficacy Questionnaire; RMDQ, Roland‐Morris Disability Questionnaire; SCL‐90h, Symptom Checklist 90, subscale hostility; SF‐36 MH, Short Form 36, subscale mental health; SF‐36: SF, Short Form 36, subscale social functioning.

^a^ *N* represents the number of cohorts.

^b^ The re‐expression of the median pre‐post effect size on a familiar instrument was obtained by multiplying the effect size by the weighted standard deviation of all post‐intervention scores from that instrument.

^c^ The pattern reflects the development of an outcome over three time points (pre, post‐final follow‐up). An upward slope between two points indicates a statistically significant favorable effect (i.e. the lower limit of the 95% CI of the ES > 0); A straight line represents no effect (i.e. the lower limit of the 95%CI of the ES < 0 and the upper limit > 0); a downward slope represents a statistically significant unfavorable effect (i.e. the upper limit of the 95% CI of the ES < 0).

^d^ Direction of effect: + represents a statistically significant effect from pre to final follow‐up; +/− represents no effect; − represents a statistically significant unfavorable effect from pre to final follow‐up. No range, only one study included.

* *p* > .05.

** *p* > .01.

*** *p* > .001.

The general trend across all outcomes indicates a statistically significant favourable effect of time in 85% of the pre‐post effect sizes. This is reflected in a positive median effect size (median SMC = 0.63, range = −0.21 to 4.93). Fifteen percent of the effect sizes show no pre‐post effects and there were no statistically significant unfavourable effects. For all cohorts that included a measurement at pre, post and follow‐up time points, a pattern with a significant pre‐post effect that is maintained at follow‐up was found in 79 (51%) of the cases. Twenty‐three patterns (15%) indicated a favourable pre‐post effect that further improved at follow‐up. A triangular relapse pattern was found in 31 (20%) of the calculated effect sizes. Two patterns (1%) showed no effect from pre to post, but a positive effect from post to follow‐up and 17 outcomes (11%) did not show any effect from pre to post or from post to follow‐up. Four outcomes (3%) showed no pre‐post effect, but an unfavourable effect from post to follow‐up. Finally, the dataset did not contain any pattern with statistically significant unfavourable pre‐post results. Not all studies included a post‐treatment measure, which explains why the pre to final follow‐up evaluations include more effect sizes than in the pattern analysis. The effect of time from pre to final follow‐up was favourable for 174 (76%) of the effect sizes. Fifty‐one effect sizes (22%) did not indicate an effect and four effect sizes (2%) showed an unfavourable effect over time. In the multimedia appendix, we provided time series plots where we standardized each outcome measure on a scale from 0 to 100 (percentage of maximum score of the measurement instrument) and plotted the development of each cohort over time using all available data points.

#### 3.2.5 Heterogeneity in outcomes

The Cochrane's *Q*‐tests for all outcomes at each of the three contrasts were significant, except for self‐efficacy at post‐follow‐up. For all other contrasts, this indicates that the null hypotheses that these studies are evaluating the same effect were rejected (Higgins & Thompson, 2002). In addition, only except for self‐efficacy at post‐follow (*I* ^2^ = 0%), all values were considerably high, with the majority of the values over 90%. These analyses support our decision to refrain from pooling the effect sizes. Rather, multiple different patient, study or interventions factors may account for this variability. The self‐efficacy post‐follow‐up contrasts indicate a stable maintenance pattern across studies.

## 4 DISCUSSION

### 4.1 Summary of findings

Our first objective was to investigate the development over time of patients who participated in IMPT programmes. The results indicate that the majority of the patient cohorts significantly improved from pre to post‐treatment. Importantly, this was mostly maintained at final follow‐up, which is in contrast to typical triangular relapse patterns that have been observed in other health behaviour change efforts (Wood & Neal, 2016). Although the results indicate that pre‐post effects of IMPT are generally maintained over time, the possibility of relapse for individual patients should not be neglected. Closer inspection of the distribution of individual cohort data, such as the post to follow‐up physical functioning data of Silvemark et al. (2014) (SMD = 0.06, 95% CI = −0.15 to 0.28), reveals that 47% of the patients show a decrease in physical functioning, assuming normally distributed data and a pre‐test–post‐test correlation of *r* = 0.59. To increase the accuracy of these rudimentary estimates, publishing the datasets along with the study, would allow for more detailed analyses on patient relapse across studies. This is especially relevant when taking into account that IMPT programmes are often considered as treatment of last resort (Jeffery et al., 2011).

Our second objective was to explore the study, patient, intervention and outcome heterogeneity of the included cohorts. In line with our expectations and with previous studies, we observed substantial methodological and statistical heterogeneity despite overlapping theoretical foundations, such as the biopsychosocial model (Geneen et al., 2017; Guzmán et al., 2001; Waterschoot et al., 2014). This heterogeneity can be explained by different policies, cultures, resources, and research traditions that have been influencing these treatment programmes over time (Kaiser et al., 2017, 2018). However, to our knowledge, the current study is the first attempt to extract and categorize the individual treatment modalities of IMPT programmes to assess the treatment content heterogeneity in these programmes. Despite a common heritage, the results of this assessment indicate that the included interventions do not share an equal underlying effect. Rather, interventions generally consist of a unique collection of multiple different action mechanisms that are generally not explicitly described.

### 4.2 Strengths and limitations

We encountered several problems regarding the interpretation of the study data. First, for the majority of the cohorts, we identified a risk of bias, which negatively influences the validity of our results. Especially the study attrition rates, indicating that a substantial minority dropped out of the programme or discontinued participation, introduce significant non‐response bias. Furthermore, incomplete reporting of the intervention and its outcomes remains an issue. To increase accuracy of reporting as well as improved understanding of how a particular IMPT programme may benefit patients, we restate the recommendation by Williams et al. (2012) to provide a clear rationale for that particular set of treatment components and to test this by including process measures (eg. Nicholas et al., 2014), instead of generally referring to a biopsychosocial approach. A practical tool that supports clear reporting is the TIDieR checklist, which includes clear guidance for reporting study rationale and action mechanisms (Hoffmann et al., 2014). Beyond the investigation of treatment benefits and harms of IMPT a standardized level of comparison, e.g. a core outcome set (Williamson et al., 2017), is required for harmonisation of outcome assessment and supporting detailed meta‐analyses. Heterogeneous outcome assessment in the context of IMPT has been consistently reported (Deckert et al., 2016; Kamper et al., 2014; Waterschoot et al., 2014). Involving the patient perspective in defining helpful treatment approaches is generally recommended for such actions (Williamson et al., 2017). For IMPT an international initiative has developed a core outcome set for a domain set of outcomes, comprising the biopsychosocial impact of chronic pain in patients undergoing IMPT (Kaiser et al., 2018). Implementing such recommendations would help also to reduce reporting bias and enhance reporting quality of clinical trials (Williamson et al., 2017). On a smaller scale, the successful implementation of similar initiatives has resulted in improved collaboration between healthcare services and a homogeneous dataset (Tardif et al., 2017). It should be noted that such initiatives are either shaped by national requirements and resources, commonly organized in national registries, or aim for international application in clinical trials (Kaiser et al., 2018). An important future challenge is to harmonize these approaches in order to achieve results in both objectives. Second, the categorisation of treatment modalities is likely to contain erroneous interpretations, either due to incomplete reporting or to misinterpretation during the data extraction. Moreover, it is important to realize that the categories for the treatment modalities, the classification of tailoring and what constitutes as an IMPT programme remain arbitrary and leave room for discussion and further refinement in future studies. To provide transparency regarding our procedures and choices, we published all data extraction forms in the online multimedia appendix. Third, the calculation of pre‐post effect sizes in meta‐analyses is under debate (Cuijpers et al., 2017; Kösters, 2017). We realize that pre‐post effects should not be considered as a valid method for demonstrating a treatment effect. However, the current analysis does provide an indication of the change over time of patients' wellbeing on several key outcomes, which is considered particularly useful from a clinician's perspective (Kösters, 2017). Of notice is that the suggested overestimation of effect size has not been observed in large comparison studies (Anglemyer et al., 2014; Benson & Hartz, 2000; Concato et al., 2000). On the contrary, less heterogeneity was found in observational studies compared to RCTs. A possible explanation was that cohorts within case series potentially better represent the population at risk and tailor treatment to specific patients, compared to RCTs with specific inclusion criteria and standardized protocols.

### 4.3 Future directions

An opportunity to increase the lifespan and relevance of this systematic review is to develop this study into a living systematic review. The main characteristic of this type of review is that it will be continuously updated when new evidence becomes available (Elliott et al., 2017). In addition, living systematic reviews often include an online platform where datasets and data analysis syntaxes are publicly available, which may decrease duplicate work (Thomas et al., 2017). This helps to decrease the evidence to practice gap, but also to stimulate collaboration and data‐sharing (Elliott et al., 2014). Data validation by authors of the included studies, improved analyses techniques as well as semi‐automated search and data extraction procedures are among the possibilities of such an initiative (Bannach‐Brown et al., 2019; Thomas et al., 2017). The current multimedia appendix has been developed to accommodate future updates, which will facilitate this transition.

## 5 CONCLUSIONS

In the past five decades, pain management programmes evolved from attempts to coordinate various disciplines in managing chronic pain to comprehensive interdisciplinary multimodal interventions that help patients to optimize their daily life functioning and their overall wellbeing. This study shows that participation in an IMPT programme is associated with considerable improvements in physical and psychological wellbeing that are generally maintained at follow‐up. The current study also revealed that despite common roots these programmes show substantial heterogeneity with respect to dose and treatment content, which suggests different viewpoints on how to optimally design an IMPT intervention. To discuss these differences and learn from this variability, we recommend to improve the precision of describing the intervention rationale and to test the proposed mechanisms by which the intervention is expected to benefit the patient. Finally, we believe that regular updates of this review may support the critical monitoring of future developments of IMPT programmes, the possibility to correct for data extraction errors and the comparison of different treatment approaches. A living systematic review approach provides the potential to accommodate this.

## CONFLICTS OF INTEREST

There is no conflict of interest.

## AUTHOR CONTRIBUTIONS

Stefan Elbers, Ulrike Kaiser, Harriët Wittink and Rob Smeets conceived of the presented idea. Jos Kleijnen and Stefan Elbers developed the search strategy. Stefan Elbers, SK, Ulrike Kaiser and Jan Pool screened the studies and extracted the data. Harriët Wittink and Rob Smeets supervised the study. Stefan Elbers and Sophie Konings developed the online multimedia appendix. All authors discussed the results and contributed to the final version of the manuscript.

## Supporting information

Supplementary Material

Supplementary Material

Supplementary Material
