# Supplementary material for: Longitudinal outcome evaluations of Interdisciplinary Multimodal Pain Treatment programmes for patients with chronic primary musculoskeletal pain: A systematic review and meta‐analysis
Source: Eur J Pain. 2021 Nov 5;26(2):310–35. doi: 10.1002/ejp.1875 (PMC9297911; doi:10.1002/ejp.1875)
Supplement: Supplementary file 1 — Supplementary Material [file EJP-26-310-s003.pdf]

**MEDLINE via OVID**	
Patient	
Specific intervention terms with multiple professions assumed	
Intervention terms (general)	
Terms related to interdisciplinary care	
Outcome / study type	
Limitations on publication type	
**1**	exp back pain/ or exp chronic pain/ or exp musculoskeletal pain/ or exp neck pain/ or exp fibromyalgia/
**2**	"chronic pain*".ti,ab,kw.
**3**	"persistent pain*".ti,ab,kw.
**4**	fibromyalgia.ti,ab,kw.
**5**	"back pain".ti,ab,kw.
**6**	"musculoskeletal pain".ti,ab,kw.
**7**	"neck pain".ti,ab,kw.
**8**	1 OR 2 OR 3 OR 4 OR 5 OR 6 OR 7
**9**	exp Pain Clinics/
**10**	"acceptance and commitment therap*".ti,ab,kw.
**11**	"functional restoration".ti,ab,kw.
**12**	"graded activity".ti,ab,kw.
**13**	"graded exposure".ti,ab,kw.
**14**	"exposure in vivo".ti,ab,kw.
**15**	"pain facilit*".ti,ab,kw.
**16**	"pain center*".ti,ab,kw.
**17**	"cognitive behavio*".ti,ab,kw.
**18**	"psychosocial intervention*".ti,ab,kw.
**19**	"psychosocial training".ti,ab,kw.
**20**	"problem solving".ti,ab,kw.
**21**	"pain treatment services".ti,ab,kw.
**22**	"chronic pain treatment".ti,ab,kw.
**23**	"pain management".ti,ab,kw.
**24**	9 or 10 or 11 or 12 or 13 or 14 or 15 or 16 or 17 or 18 or 19 or 20 or 21 or 22 or 23
**25**	exp Rehabilitation/
**26**	exp "Exercise Therapy"/
**27**	treatment.ti,ab,kw.
**28**	therapy.ti,ab,kw.
**29**	management.ti,ab,kw.
**30**	"pain service*".ti,ab,kw.
**31**	rehabilitation.ti,ab,kw.
**32**	25 or 26 or 27 or 28 or 29 or 30 or 31
**33**	Interprofessional.ti,ab,kw.
**34**	exp "Patient care team"/
**35**	interdisciplinary.ti,ab,kw.
**36**	multidisciplinary.ti,ab,kw.
**37**	multimodal.ti,ab,kw.
**38**	"integrated care".ti,ab,kw.

**39**	"comprehensive care".ti,ab,kw.
**40**	33 or 34 or 35 or 36 or 37 or 38 or 39
**41**	Exp self-efficacy/
**42**	Exp activities of daily living/
**43**	Exp quality of life/
**44**	Exp Follow-up studies/
**45**	Exp prospective studies/
**46**	"Treatment Outcome".ti,ab,kw.
**47**	"Clinical Effectiveness".ti,ab,kw.
**48**	"Patient-Relevant Outcome*".ti,ab,kw.
**49**	"Clinical Efficacy".ti,ab,kw.
**50**	"Treatment Effectiveness".ti,ab,kw.
**51**	"Treatment Efficacy".ti,ab,kw.
**52**	"Rehabilitation Outcome".ti,ab,kw.
**53**	"Health Related quality of life".ti,ab,kw.
**54**	"Quality of life".ti,ab,kw.
**55**	"Life Quality".ti,ab,kw.
**56**	"Physical function*".ti,ab,kw.
**57**	"Emotional function*".ti,ab,kw.
**58**	anger.ti,ab,kw.
**59**	"Self-efficacy".ti,ab,kw.
**60**	"pain intensity".ti,ab,kw.
**61**	"disability".ti,ab,kw.
**62**	"follow-up".ti,ab,kw.
**63**	"long-term".ti,ab,kw.
**64**	"longitudinal".ti,ab,kw.
**65**	"Prospective".ti,ab,kw.
**66**	"Cost effectiveness".ti,ab,kw.
**67**	41 or 42 or 43 or 44 or 45 or 46 or 47 or 48 or 49 or 50 or 51 or 52 or 53 or 54 or 55 or 56 or 57 or 58 or 59 or 60 or 61 or 62 or 63 or 64 or 65 or 66
**68**	32 and 40
**69**	24 or 68
**70**	8 and 69
**71**	67 and 70
**72**	(address or autobiography or bibliography or biography or "classical article" or comment or congress or "consensus development conference" or dictionary or directory or editorial or "expression of concern" or "government document" or guideline or "interactive tutorial" or interview or lecture or "legal case" or legislation or letter or news or "newspaper article" or "observational study, veterinary " or "patient education handout" or "periodical index" or "personal narrative" or portrait or "scientific integrity review" or "technical report" or "twin study" or "video-audio media" or "webcasts").pt.
**73**	71 not 72
	**Hits on 08-05-2019: 4478**
	**Hits on 07-05-2020: 329**

**PsycINFO via EBSCO**	
Patient	
Specific intervention terms with multiple professions assumed	
Intervention terms (general)	
Terms related to interdisciplinary care	
Outcome / study type	
Limitations on publication type	
Terms that are not available in Thesaurus: Musculosketal pain; neck pain.	
**1**	DE "back pain" OR DE "chronic pain" OR DE "fibromyalgia"
**2**	TI "chronic pain" OR AB "chronic pain"
**3**	TI "persistent pain" OR AB "persistent pain"
**4**	TI "fibromyalgia" OR AB "fibromyalgia"
**5**	TI "back pain" OR AB "back pain"
**6**	TI "musculoskeletal pain" OR AB "musculoskeletal pain"
**7**	TI "neck pain" OR AB "neck pain"
**8**	S1 OR S2 OR S3 OR S4 OR S5 OR S6 OR S7
**9**	DE "pain management"
**10**	DE "acceptance and commitment therapy"
**11**	DE "in vivo exposure"
**12**	DE "cognitive behavior therapy"
**13**	DE "psychosocial rehabilitation"
**14**	DE "biopsychosocial approach"
**15**	TI ("acceptance and commitment therap*") OR AB ("acceptance and commitment therap*")
**16**	TI "functional restoration" OR AB "functional restoration"
**17**	TI "graded activity" OR AB "graded activity"
**18**	TI "graded exposure" OR AB "graded exposure"
**19**	TI "exposure in vivo" OR AB "exposure in vivo"
**20**	TI "pain facilit*" OR AB "pain facilit*"
**21**	TI "pain center*" OR AB "pain center*"
**22**	TI "cognitive behavio*" OR AB "cognitive behavio*"
**23**	TI "psychosocial intervention*" OR AB "psychosocial intervention*"
**24**	TI "psychosocial training" OR AB "psychosocial training"
**25**	TI "problem solving" OR AB "problem solving"
**26**	TI "pain treatment services" OR AB "pain treatment services"
**27**	TI "chronic pain treatment" OR AB "chronic pain treatment"
**28**	TI "pain management" OR AB "pain management"
**29**	S9 OR S10 OR S11 OR S12 OR S13 OR S14 OR S15 OR S16 OR S17 OR S18 OR S19 OR S20 OR S21 OR S22 OR S23 OR S24 OR S25 OR S26 OR S27 OR S28
**30**	DE "rehabilitation"
**31**	DE "rehabilitation centers"
**32**	DE "movement therapy"
**33**	TI "treatment" AND AB "treatment"
**34**	TI "therapy" AND AB "therapy"
**35**	TI "management" AND AB "management"

**36**	TI "pain service" AND AB "pain service"
**37**	TI "rehabilitation" AND AB "rehabilitation"
**38**	S30 S31 OR S32 OR S33 OR S34 OR S35 OR S36 OR S37
**39**	TI interprofessional AND AB interprofessional
**40**	TI interdisciplinary AND AB interdisciplinary
**41**	TI multidisciplinary AND AB multidisciplinary
**42**	TI multimodal AND AB multimodal
**43**	TI "integrated care" AND AB "integrated care"
**44**	TI "comprehensive care" AND AB "comprehensive care"
**45**	S39 OR S40 OR S41 OR S42 OR S43 OR S44
**46**	DE "activities of daily living"
**47**	DE "self-efficacy"
**48**	DE "quality of life"
**49**	DE "health related quality of life"
**50**	DE "posttreatment followup"
**51**	DE "followup studies"
**52**	DE "prospective studies"
**53**	TI "treatment outcome" OR AB "treatment outcome"
**54**	TI "clinical effectiveness" OR AB "clinical effectiveness"
**55**	TI "patient-relevant outcomes" OR AB "patient-relevant outcomes"
**56**	TI "clinical efficacy" OR AB "clinical efficacy"
**57**	TI "treatment effectiveness" OR AB "treatment effectiveness"
**58**	TI "treatment efficacy" OR AB "treatment efficacy"
**59**	TI "rehabilitation outcome" OR AB "rehabilitation outcome"
**60**	TI "health related quality of life" OR AB "health related quality of life"
**61**	TI "quality of life" OR AB "quality of life"
**62**	TI "life quality" OR AB "life quality"
**63**	TI "physical function*" OR AB "physical function*"
**64**	TI "emotional function*" OR AB "emotional function*"
**65**	TI anger OR AB anger
**66**	TI "self-efficacy" OR AB "self-efficacy"
**67**	TI "pain-intensity" OR AB "pain-intensity"
**68**	TI disability OR AB disability
**69**	TI "follow-up" OR AB "follow-up"
**70**	TI "followup" OR AB "followup"
**71**	TI "long-term" OR AB "long-term"
**72**	TI longitudinal OR AB longitudinal
**73**	TI prospective OR AB prospective
**74**	TI "cost effectiveness" OR AB "cost effectiveness"
**75**	S44 OR S45 OR S46 OR S47 OR S48 OR S49 OR S50 OR S51 OR S52 OR S53 OR S54 OR S55 OR S56 OR S57 OR S58 OR S59 OR S60 OR S61 OR S62 OR S63 OR S64 OR S65 OR S66 OR S67 OR S68 OR S69 OR S70 OR S71 OR S72 OR S73 OR S74
**76**	S38 AND S45
**77**	S29 OR S76
**78**	S8 AND S77

**79**	S75 AND S78
**80**	Limiters - Publication Type: All Books, Authored Book, Edited Book, Encyclopedia, Electronic Collection
**81**	S79 NOT S80
	Hits on 09-05-2019: 3175
	Hits on 07-05-2020: 153

**EMBASE via OVID**	
Patient	
Specific intervention terms with multiple professions assumed	
Intervention terms (general)	
Terms related to interdisciplinary care	
Outcome / study type	
Limitations on publication type	
**1**	Exp chronic pain / or exp musculoskeletal pain/ or exp fibromyalgia/
**2**	"chronic pain*".ti,ab,kw.
**3**	"persistent pain*".ti,ab,kw.
**4**	fibromyalgia.ti,ab,kw.
**5**	"back pain".ti,ab,kw.
**6**	"musculoskeletal pain".ti,ab,kw.
**7**	"neck pain".ti,ab,kw.
**8**	1 OR 2 OR 3 OR 4 OR 5 OR 6 OR 7
**9**	Exp pain clinic/
**10**	Exp problem solving/
**11**	Exp cognitive therapy/
**12**	Exp psychosocial rehabilitation/
**13**	"acceptance and commitment therap*".ti,ab,kw.
**14**	"functional restoration".ti,ab,kw.
**15**	"graded activity".ti,ab,kw.
**16**	"graded exposure".ti,ab,kw.
**17**	"exposure in vivo".ti,ab,kw.
**18**	"pain facilit*".ti,ab,kw.
**19**	"pain center*".ti,ab,kw.
**20**	"cognitive behavio*".ti,ab,kw.
**21**	"psychosocial intervention*".ti,ab,kw.
**22**	"psychosocial training".ti,ab,kw.
**23**	"problem solving".ti,ab,kw.
**24**	"pain treatment services".ti,ab,kw.
**25**	"chronic pain treatment".ti,ab,kw.
**26**	"pain management".ti,ab,kw
**27**	9 or 10 or 11 or 12 or 13 or 14 or 15 or 16 or 17 or 18 or 19 or 20 or 21 or 22 or 23 or 24 or 25 or 26
**28**	exp Rehabilitation/
**29**	exp Exercise Therapy/
**30**	treatment.ti,ab,kw.
**31**	therapy.ti,ab,kw.
**32**	management.ti,ab,kw.
**33**	"pain service*".ti,ab,kw.
**34**	rehabilitation.ti,ab,kw.
**35**	28 or 29 or 30 or 31 or 32 or 33 or 34
**36**	Exp multidisciplinary team/
**37**	exp Patient care team/
**38**	Interprofessional.ti,ab,kw.

**39**	interdisciplinary.ti,ab,kw.
**40**	multidisciplinary.ti,ab,kw.
**41**	multimodal.ti,ab,kw.
**42**	"integrated care".ti,ab,kw.
**43**	"comprehensive care".ti,ab,kw.
**44**	36 or 37 or 38 or 39 or 40 or 41 or 42 or 43
**45**	Exp self-efficacy/
**46**	Exp daily life activity/
**47**	Exp quality of life/
**48**	Exp Follow-up studies/
**49**	Exp prospective studies/
**50**	"Treatment Outcome".ti,ab,kw.
**51**	"Clinical Effectiveness".ti,ab,kw.
**52**	"Patient-Relevant Outcome*".ti,ab,kw.
**53**	"Clinical Efficacy".ti,ab,kw.
**54**	"Treatment Effectiveness".ti,ab,kw.
**55**	"Treatment Efficacy".ti,ab,kw.
**56**	"Rehabilitation Outcome".ti,ab,kw.
**57**	"Health Related quality of life".ti,ab,kw.
**58**	"Quality of life".ti,ab,kw.
**59**	"Life Quality".ti,ab,kw.
**60**	"Physical function*".ti,ab,kw.
**61**	"Emotional function*".ti,ab,kw.
**62**	anger.ti,ab,kw.
**63**	"Self-efficacy".ti,ab,kw.
**64**	"pain intensity".ti,ab,kw.
**65**	"disability".ti,ab,kw.
**66**	"follow-up".ti,ab,kw.
**67**	"long-term".ti,ab,kw.
**68**	"longitudinal".ti,ab,kw.
**69**	"Prospective".ti,ab,kw.
**70**	"Cost effectiveness".ti,ab,kw.
**71**	45 or 46 or 47 or 48 or 49 or 50 or 51 or 52 or 53 or 54 or 55 or 56 or 57 or 58 or 59 or 60 or 61 or 62 or 63 or 64 or 65 or 66 or 67 or 68 or 69 or 70
**72**	35 and 44
**73**	27 or 72
**74**	8 and 73
**75**	71 and 74
**76**	(conference abstract or conference paper or conference review or editorial or letter or note).pt.
**77**	75 not 76
	**Hits on 08-05-2019: 9495**
	**Hits on 07-05-2020: 1184**
	**chunk strategy for export (max 5000 citations)**<br>Limit 77 to yr="1883 – 2011" [chunk 1]

	77 not 78 [chunk 2]

**CINAHL via EBSCO**<br>Username: stefanelbers | Password: IMPTreview1!	
Patient	
Specific intervention terms with multiple professions assumed	
Intervention terms (general)	
Terms related to interdisciplinary care	
Outcome / study type	
Limitations on publication type	
Terms that are not available in Thesaurus: Musculosketal pain; neck pain.	
**1**	MH("back pain+" or "chronic pain+" or "fibromyalgia+")
**2**	TI "chronic pain" OR AB "chronic pain"
**3**	TI "persistent pain" OR AB "persistent pain"
**4**	TI "fibromyalgia" OR AB "fibromyalgia"
**5**	TI "back pain" OR AB "back pain"
**6**	TI "musculoskeletal pain" OR AB "musculoskeletal pain"
**7**	TI "neck pain" OR AB "neck pain"
**8**	S1 OR S2 OR S3 OR S4 OR S5 OR S6 OR S7
**9**	MH "Cognitive Therapy+"
**10**	MH "Pain Management"
**11**	MH "pain clinics"
**12**	TI ("acceptance and commitment therap*") OR AB ("acceptance and commitment therap*")
**13**	TI "functional restoration" OR AB "functional restoration"
**14**	TI "graded activity" OR AB "graded activity"
**15**	TI "graded exposure" OR AB "graded exposure"
**16**	TI "exposure in vivo" OR AB "exposure in vivo"
**17**	TI "pain facilit*" OR AB "pain facilit*"
**18**	TI "pain center*" OR AB "pain center*"
**19**	TI "cognitive behavio*" OR AB "cognitive behavio*"
**20**	TI "psychosocial intervention*" OR AB "psychosocial intervention*"
**21**	TI "psychosocial training" OR AB "psychosocial training"
**22**	TI "problem solving" OR AB "problem solving"
**23**	TI "pain treatment services" OR AB "pain treatment services"
**24**	TI "chronic pain treatment" OR AB "chronic pain treatment"
**25**	TI "pain management" OR AB "pain management"
**26**	S9 OR S10 OR S11 OR S12 OR S13 OR S14 OR S15 OR S16 OR S17 OR S18 OR S19 OR S20 OR S21 OR S22 OR S23 OR S24 OR S25
**27**	MH rehabilitation+
**28**	MH "Multidisciplinary care team"
**29**	TI "treatment" AND AB "treatment"
**30**	TI "therapy" AND AB "therapy"
**31**	TI "management" AND AB "management"
**32**	TI "pain service" AND AB "pain service"
**33**	TI "rehabilitation" AND AB "rehabilitation"
**34**	S27 OR S28 OR S29 OR S30 S31 OR S32 OR S33

**35**	MH "Combined Modality Therapy+"
**36**	TI interprofessional AND AB interprofessional
**37**	TI interdisciplinary AND AB interdisciplinary
**38**	TI multidisciplinary AND AB multidisciplinary
**39**	TI multimodal AND AB multimodal
**40**	TI "integrated care" AND AB "integrated care"
**41**	TI "comprehensive care" AND AB "comprehensive care"
**42**	S35 OR S36 OR S37 OR S38 OR S39 OR S40 OR S41
**43**	MH self-efficacy
**44**	MH "quality of life+"
**45**	MH "prospective studies+"
**46**	TI "treatment outcome" OR AB "treatment outcome"
**47**	TI "clinical effectiveness" OR AB "clinical effectiveness"
**48**	TI "patient-relevant outcomes" OR AB "patient-relevant outcomes"
**49**	TI "clinical efficacy" OR AB "clinical efficacy"
**50**	TI "treatment effectiveness" OR AB "treatment effectiveness"
**51**	TI "treatment efficacy" OR AB "treatment efficacy"
**52**	TI "rehabilitation outcome" OR AB "rehabilitation outcome"
**53**	TI "health related quality of life" OR AB "health related quality of life"
**54**	TI "quality of life" OR AB "quality of life"
**55**	TI "life quality" OR AB "life quality"
**56**	TI "physical function*" OR AB "physical function*"
**57**	TI "emotional function*" OR AB "emotional function*"
**58**	TI anger OR AB anger
**59**	TI "self-efficacy" OR AB "self-efficacy"
**60**	TI "pain-intensity" OR AB "pain-intensity"
**61**	TI disability OR AB disability
**62**	TI "follow-up" OR AB "follow-up"
**63**	TI "followup" OR AB "followup"
**64**	TI "long-term" OR AB "long-term"
**65**	TI longitudinal OR AB longitudinal
**66**	TI prospective OR AB prospective
**67**	TI "cost effectiveness" OR AB "cost effectiveness"
**68**	S43 OR S44 OR S45 OR S46 OR S47 OR S48 OR S49 OR S50 OR S51 OR S52 OR S53 OR S54 OR S55 OR S56 OR S57 OR S58 OR S59 OR S60 OR S61 OR S62 OR S63 OR S64 OR S65 OR S66 OR S67
**69**	S34 AND S42
**70**	S26 OR S69
**71**	S8 AND S70
**72**	S68 AND S71
**73**	(Abstract OR Accreditation OR Algorithm OR Anecdote OR Audiovisual OR Bibliography OR Biography OR Book OR Book chapter OR Brief item OR Care plan OR Cartoon OR CEU OR Classification term OR Code of ethics OR Computer program OR Consumer patient teaching materials OR Critical path OR Diagnostic images OR Drugs OR Editorial OR Equations formulas OR Exam questions OR Forms OR Games OR Glossary OR Historical

	material OR Interview OR Journal description OR Legal cases OR Letter OR Masters thesis OR Nurse practice acts OR Nursing diagnoses OR Obituary OR Overall OR Pamphlet OR Pamphlet chapter OR Pictorial OR Poetry OR Practice acts OR Practice guidelines OR OR Proceedings OR Questionnaire scale OR Questions and answers OR Research instrument OR Research term definition OR Response OR Software OR Standards OR Tables charts OR Teaching materials OR Tracings OR Website)
**74**	S72 NOT S73
	HITS ON 09-05-2019: 1110
	HITS ON 07-05-2020: 227 (limit: publication date 2019-2020)
