# Supplementary material for: Longitudinal outcome evaluations of Interdisciplinary Multimodal Pain Treatment programmes for patients with chronic primary musculoskeletal pain: A systematic review and meta‐analysis
Source: Eur J Pain. 2021 Nov 5;26(2):310–35. doi: 10.1002/ejp.1875 (PMC9297911; doi:10.1002/ejp.1875)
Supplement: Supplementary file 2 — Supplementary Material [file EJP-26-310-s001.pdf]

Data extraction form

Fill in one form for each intervention cohort. This form can also accommodate a control group. In case of more than 1 control groups, choose the most active control group.

* Required

1. Email address *

General study information

2. author

3. year

4. id

5. number_of_cohorts

   How many cohorts meet the intervention conditions of this review

6. cohort_id

   Number of the cohort that you now processing

7. cohort_name

Brief name of this intervention cohort

8. assessor

Mark only one oval.

- SE
- SK
- UK
- SKSE
- MK
- HW

extra cohort

9. first_cohort_description

Is this the first cohort that you are describing?: In case of 'no', you will be forwarded to the participants section. (if only 1 cohort is present in the study choose yes)

Mark only one oval.

- Yes
- No *Skip to question 19*

Population, setting and design

10. nationality

Population description (nationality)

11. method_patient_recruitment

Method of patient recruitment; Recruitment concerns how people get into the study (not how people get into the intervention program)

patient_inclusion

Hier vallen 'patient group', 'minimum pain duration' en 'referral' onder

12. patient_group

For example; Low back pain, Chronic pain, Fibromyalgia......

13. minimum_pain_duration

The length of time as in the author's inclusion criterion (e.g. lbp for a minimum of three months)

14. referral

Referral has to do with the way in which people enter the treatment program. For example, General practitioners, specialists, primary healthcare practictioners.....

15. patient_exclusion_criteria

16. total_inclusion_criteria

list all remaining inclusion criteria (numbered) below

17. study_design

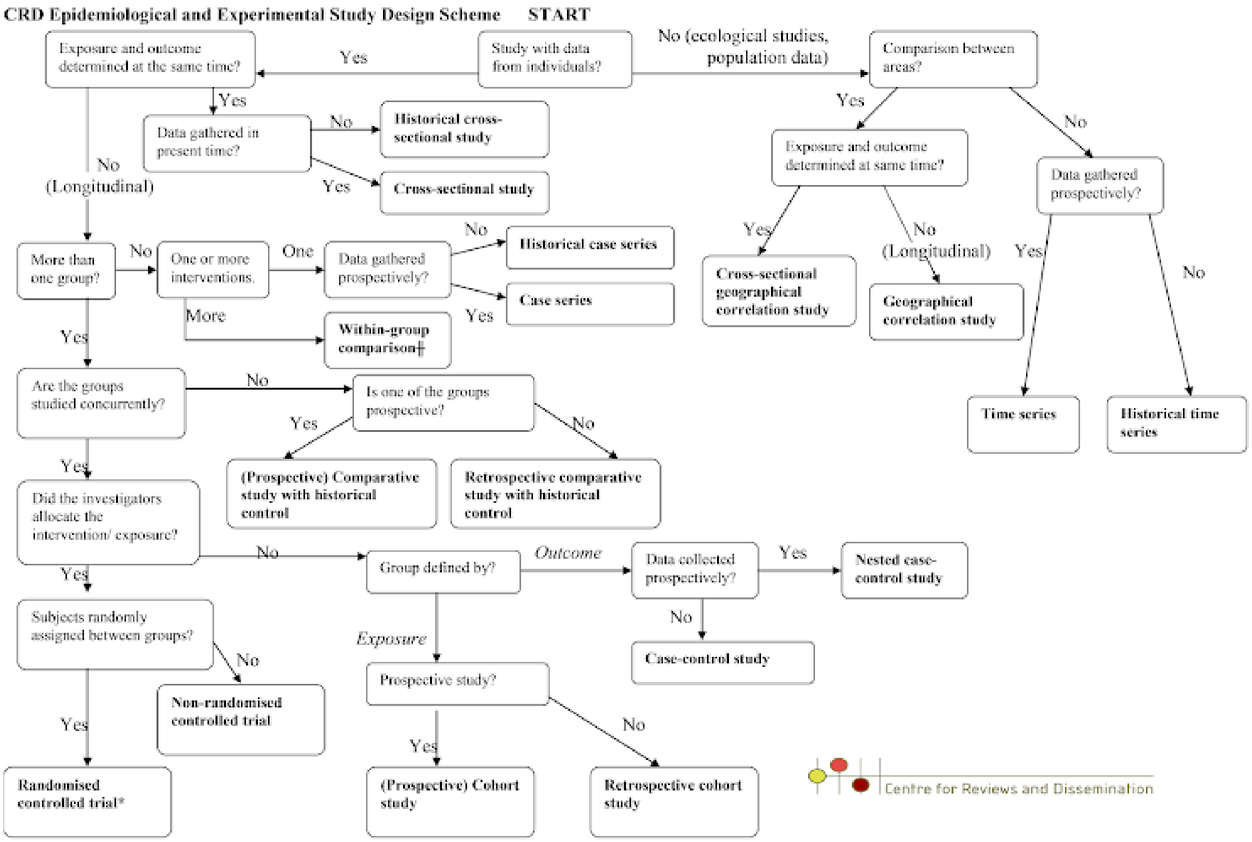

*Other types of randomised trials: cross-over randomised controlled trial. ╫ This has previously been referred to as a before and after study within CRD report 6.

Mark only one oval.

- ⬭ RCT
- ⬭ N-RCT
- ⬭ case series
- ⬭ prospective cohort study
- ⬭ retrospective cohort study
- ⬭ historical case series
- ⬭ prospective comparative study with historical control
- ⬭ retrospective comparative study with historical control
- ⬭ Other: ____________________

18. sample_size

total study sample size

____

participants

19. sample_size_pre

20. sample_size_post

21. sample_size_fu

Sample size (latest follow-up)

22. demographics_population_or_cohort

Choose the correct option for the demographics below in case that both answers are correct, choose 'study cohort'. If the study contains 1 cohort, choose 'both'.

Mark only one oval.

- demographics relate to the total study population
- demographics relate to the specific study cohort
- both

23. age_m

Mean age of the cohort (Display with a point between decimals and whole numbers)

24. age_sd

the standard deviation associated with the mean age (Display with a point between decimals and whole numbers)

25. female_gender

%female (Display with a point between decimals and whole numbers)

26. pain_duration_months_m

Mean pain duration in months of the cohort(baseline) (Display with a point between decimals and whole numbers)

27. pain_duration_sd

The standard deviation associated with the mean pain duration of the cohort (baseline) (Display with a point between decimals and whole numbers)

intervention_groups

why

28. study_rationale

describe study rationale: you can leave this blank if you already described this for another cohort within this study.

29. study_objective

describe study objective: you can leave this blank if you already described this for another cohort within this study.

30. treatment_aim

describe treatment aim

what

31. materials

Materials: Describe any physical or informational materials used in the intervention, including those provided by participants or used in the intervention, including those provided by participants or used in the intervention delivery or in training of intervention providers. Provide information on where the materials can be accessed (e.g. online, appendix, intervention protocols).

32. procedures

Procedures: Describe all procedures, activities and/or processes used in the intervention, including any enabling or support activities.

33. treatment_modalities

Please categorize the aforementioned procedures below

1. ED: education
2. EX: Exercise
-Muscle strengthening
- (Muscle) stretching
- Aerobic activity
- Swimming pool activities (+hydrotherapy)
- Rhythm and movement exercise
- Mobility/stability exercise
- homework exercises
- Nordic walking
-warming and stretching exercises
- core training
- balance/ coordination training
-sports
-work simulation/ hardening;
-posture maintenance
-functional exercises
3. GA: graded activity
- graded activity
- behavioral exercise therapy
-Graded Exercise Therapy
-Pacing (+ starting at 80%, gradually increasing)
-Operant-behavioral graded activity training
4. BT: (cognitive behavioral therapy)
- CBT
- Cognitive treatment
- ACT
- Problem solving
- Rational emotive therapy (RET)
-Behavior oriented counseling
- Graded Exposure
5. relaxation
- breathing techniques
- progressive relaxation
- muscle relaxation
- autogenic training
- mindfulness training
-applied relaxation
-cognitive relaxation
-physical relaxation
-massage therapy
-electromyography biofeedback training: enhance muscle relaxation techniques
-vegetative stabilization
-Balneotherapy
6.PM: Pain management skills
- goal setting
- pacing
- ergonomics
- structuring daily activities
-identifing barriers to recovery/ management
-stress management skills
-behavioral home tasks
-developing adaptive regulation of daily living
7.Pharmalogical treatment (code medication withdrawal procedures as 'other')
8.Body awareness therapy
- Biofeedback
9.Workplace advice
-workplace visit (kaapa)
-ergonomic advise on the workplace
-return to work program
10. Team meetings (only include if patient is present)
11. Other

Check all that apply.

- [ ] education
- [ ] exercise
- [ ] graded activity
- [ ] (cognitive) behavioral therapy
- [ ] relaxation
- [ ] pain management skills
- [ ] pharmacological treatment
- [ ] body awareness therapy
- [ ] workplace advice
- [ ] team meetings
- [ ] Other

34. other_procedures

Please list any procedures that you could not classify

35. procedures_bps_conflict

please list any procedures that might be in conflict with a biopsychosocial orientation to chronic pain

who_provided

36. healthcare_providers

Select all involved healthcare providers

Check all that apply.

- [ ] Physician (rehabilitation, occupational, general)
- [ ] psychologist (clinical psychologist, behavioral therapist)
- [ ] physiotherapist (physical therapist)
- [ ] occupational therapist (ergo therapist)
- [ ] social worker (counsellor, advisor)
- [ ] nurse
- [ ] other

37. other_healthcare_providers

if there where other healthcare providers involved, please list each provider below:

____
____
____
____
____

mode_of_delivery

38. in_out_patient

In or outpatient treatment? (sub info of the mode of delivery)

Mark only one oval.

- ( ) inpatient
- ( ) outpatient
- ( ) inpatient and outpatient combined

39. type_of_contact

Group or individual treatment (sub info of the mode of delivery)

Mark only one oval.

- ( ) Group (>90%)
- ( ) Individual (>90%)
- ( ) Mixed

40. group_size

If applicable, please specify group size or range of de groep size

____

41. mode_of_delivery_subinfo

(sub info of the mode of delivery)

Check all that apply.

- [ ] face-to-face
- [ ] eHealth or mHealth coaching
- [ ] coaching by telephone

where

42. setting

Mark only one oval.

- ( ) hospital
- ( ) university
- ( ) treatment/rehabilitation center
- ( ) pain clinic
- ( ) community center
- ( ) primary care setting
- ( ) Other: ____________________

when_and_how_much

Describe the number of times the intervention was delivered and over what period of time including the number of sessions, their schedule, and their duration, intensity or dose.

43. time_span

Duration of the intervention in weeks

____

duration_direct_treatment_time

Total duration of direct treatment time (assume 6 hours per day if not specified)

44. hours

Duration of the intervention in hours

____

45. minutes

Duration of the intervention in minutes (min the whole hours)

____

46. duration_treatment_time_based on_estimate

was the total hours of treatment time based on an estimate?

Mark only one oval.

- ( ) yes
- ( ) no

tailoring

47. tailoring_mentioned_in_study

Did the study mention any form of tailoring?

Mark only one oval.

- ( ) Yes
- ( ) No

48. tailoring

Mark only one oval.

- ( ) Low
- ( ) Medium
- ( ) High

tailoring

49. description_tailoring

If tailoring is mentioned: describe what, why, when and how the intervention was planned to be personalized, titrated or adapted.

50. followup_sessions_provided

Was er nazorg in de zin van boostersessies, webbased interventies, telefonisch contact etc.

Mark only one oval.

- Yes
- No

51. followup_sessions_description

Specify the follow-up sessions (Specificeer de nazorg in de zin van boostersessies, webbased interventies, telefonisch contact etc.)

modifications

52. modifications_mentioned_in_study

did the study report any modifications that were made to the intervention during the study period?

Mark only one oval.

- Yes *Skip to question 53*
- No *Skip to question 54*

modifications_II

53. description_modifications

describe what, why, when and how the intervention was modified during the course of the study.

how_well

54. adherence_or_fidelity_assessed

did the study assess adherence or fidelity to the study protocol of healthcare professionals

Mark only one oval.

- Yes
- No *Skip to question 57*

how_well_II

55. description_fidelity_strategies

describe how and by whom and if any strategies were used to maintain or improve fidelity

56. changes_intervention

to what extent was the intervention delivered as planned?

control_groups

57. control_groups_present

did the study have control groups?

Mark only one oval.

- ⬭ Yes *Skip to question 58*
- ⬭ No *Skip to question 60*

control_groups_II

58. name_of_ control_group

please specify the name of the control group: if more than 1 controls groups are reported, please select the most active type of control group

59. type_of_control_group

Specify the type of control group

Mark only one oval.

- ⬭ treatment as usual
- ⬭ waiting list
- ⬭ active

outcomes

60. study_outcomes

please list all outcomes from the study: abbreviations are preferred. please use semicolons to delineate.

61. months_last_fu

please provide the time of the final follow-up measurement in months

62. measurement_of_hrqol

Does the study include are measure of HRQOL?

Mark only one oval.

- Yes
- No *Skip to question 85*

HRQOL

63. hrqol_name_measurement_instrument

hrqol: What is the name of the measurement instrument?

Mark only one oval.

- mean NHP (0-100)
- SF -36
- EuroQol-5D-3L (VAS 0-100)
- SF-12
- FIQ (0-100)
- WHOQOL-BREF: global (0-100)
- LiSat-11: life a a whole (1-6)
- RAND-36
- Other
- German Life Satisfaction Questionnaire

64. hrqol_pre_n

if seperate N is described for HRQOL, please specify. Only fill in if the N deviates from the standard N, otherwise leave blank (fill in N if the author has applied imputation due to missing)

65. hrqol_pre_m

enter the mean for HRQOL pre

66. hrqol_pre_sd

enter the SD for HRQOL pre

67. hrqol_post_n

if sepatate N is described for HRQOL at post treatment, please specify. Only fill in if the N deviates from the standard N, otherwise leave blank (fill in N if the author has applied imputation due to missing)

68. hrqol_post_m

69. hrqol_post_sd

70. hrqol_fu1_t

hrqol: Follow up 1: specify months after end of treatment

71. hrqol_fu1_n

if sepatate N is described for HRQOL at fu1, please specify. Only fill in if the N deviates from the standard N, otherwise leave blank (fill in N if the author has applied imputation due to missing)

72. hrqol_fu1_m

73. hrqol_fu1_sd

74. hrqol_measurement_after_fu1

hrqol: Are there more follow-up measurements?

Mark only one oval.

- Yes *Skip to question 75*
- No *Skip to question 85*

HRQOL_FU2

75. hrqol_fu2_t

hrqol: Follow up 2: specify months after end of treatment

76. hrqol_fu2_n

if sepatate N is described at fu2, please specify. Only fill in if the N deviates from the standard N, otherwise leave blank (fill in N if the author has applied imputation due to missing)

77. hrqol_fu2_m

78. hrqol_fu2_sd

79. hrqol_measurement_after_fu2

Mark only one oval.

- Yes
- No *Skip to question 85*

HRQOL_FU3

this is the maximum amount of follow-up moments. Please list the final measurement.

80. hrqol_fu3_t

hrqol: Follow up 3: specify months after end of treatment

81. hrqol_fu3_n

if sepatate N is described at fu3, please specify. Only fill in if the N deviates from the standard N, otherwise leave blank (fill in N if the author has applied imputation due to missing)

82. hrqol_fu3_m

83. hrqol_fu3_sd

84. hrqol_measurement_after_fu3

hrqol: Are there any more follow-up measurements that you were not able to include?

Mark only one oval.

- Yes
- No

physical_functioning

85. measurements_physical_functioning

Does the study include are measure of Physical functioning?

Mark only one oval.

- Yes
- No *Skip to question 108*

physical_functioning_II

86. pf_measurement_name

pf: What is the name of the measurement instrument?

Mark only one oval.

- NHP: PA
- SF-36 subscale Physical Functioning
- SF-36 summary scale PCS
- HFAQ
- MPI: GA (0-6)
- RAND-36 subscale Physical Functioning
- RAND-36 summary scale PCS
- COMI: function
- Other

87. pf_pre_n

if sepatate N is described for pf at pre, please specify. Only fill in if the N deviates from the standard N, otherwise leave blank (fill in N if the author has applied imputation due to missing)

88. pf_pre_m

89. pf_pre_sd

90. pf_post_n

if sepatate N is described at post, please specify. Only fill in if the N deviates from the standard N, otherwise leave blank (fill in N if the author has applied imputation due to missing)

91. pf_post_m

92. pf_post_sd

93. pf_fu1_t

pf: Follow up 1: specify months after end of treatment

94. pf_fu1_n

if sepatate N is described at fu1, please specify. Only fill in if the N deviates from the standard N, otherwise leave blank (fill in N if the author has applied imputation due to missing)

95. pf_fu1_m

96. pf_fu1_sd

97. pf_measurement_after_fu1

Mark only one oval.

- Yes *Skip to question 98*
- No *Skip to question 108*

physical_functioning_fu2

98. pf_fu2_t

pf: Follow up 2: specify months after end of treatment

99. pf_fu2_n

if sepatate N is described at fu2, please specify. Only fill in if the N deviates from the standard N, otherwise leave blank (fill in N if the author has applied imputation due to missing)

100. pf_fu2_m

101. pf_fu2_sd

102. pf_measurement_after_fu2

pf: Are there more follow-up measurements?

Mark only one oval.

- Yes
- No *Skip to question 108*

physical_functioning_fu3

this is the maximum amount of follow-up moments. Please list the final measurement.

103. pf_fu3_t

pf: Follow up 3: specify months after end of treatment

104. pf_fu3_n

if sepatate N is described at fu3, please specify. Only fill in if the N deviates from the standard N, otherwise leave blank (fill in N if the author has applied imputation due to missing)

105. pf_fu3_m

____

106. pf_fu3_sd

____

107. pf_measurement_after_fu3

pf: Are there any more follow-up measurements that you were not able to include?

Mark only one oval.

- Yes
- No

pain_interference

Note: For questions about reporting the N -> if sepatate N is described for pinter, please specify. Only fill in if the N deviates from the standard N, otherwise leave blank (fill in N if the author has applied imputation due to missing)

108. measurements_pain_interference

Does the study include are measure of pain interference?

Mark only one oval.

- Yes
- No *Skip to question 131*

pain_interference_II

109. pinter_name_measurement_instrument

Mark only one oval.

- RMDQ
- QBPDS
- MPI: pain interference (0-6)
- MPI: pain interference (0-12)
- MPI: pain interference (0-100)
- BPI
- PDI
- DRI
- ODI
- NDI
- LBPRS
- SIP
- DPQ: Daily activities
- FRI
- Other

110. pinter_pre_n

if seperate N is described for HRQOL, please specify

111. pinter_pre_m

112. pinter_pre_sd

113. pinter_post_n

114. pinter_post_m

115. pinter_post_sd

116. pinter_fu1_t
pinter: Follow up 1: specify months after end of treatment

117. pinter_fu1_n

118. pinter_fu1_m

119. pinter_fu1_sd

120. pinter_measurement_after_fu1
pinter: Are there more follow-up measurements?

Mark only one oval.

- Yes
- No *Skip to question 131*

pain_interference_fu2

121. pinter_fu2_t

pinter: Follow up 2: specify months after end of treatment

____

122. pinter_fu2_n

____

123. pinter_fu2_m

____

124. pinter_fu2_sd

____

125. pinter_measurement_after_fu2

pinter: Are there more follow-up measurements?

Mark only one oval.

- ( ) Yes
- ( ) No *Skip to question 131*

pain_interference_fu3

this is the maximum amount of follow-up moments. Please list the final measurement.

126. pinter_fu3_t

pinter: Follow up 3: specify months after end of treatment

____

127. pinter_fu3_n

____

128. pinter_fu3_m

____

129. pinter_fu3_sd

____

130. pinter_measurement_after_fu3

pinter: Are there any more follow-up measurements that you were not able to include?

Mark only one oval.

- Yes
- No

depression

Note: For questions about reporting the N -> if sepatate N is described for dep, please specify. Only fill in if the N deviates from the standard N, otherwise leave blank (fill in N if the author has applied imputation due to missing)

131. measurements_depression

Does the study include are measure of depression?

Mark only one oval.

- Yes
- No *Skip to question 154*

depression_II

132. dep_name_measurement_instrument

dep: What is the name of the measurement instrument?

Mark only one oval.

- DASS
- General Depression scale
- BDI-II
- HADS-D
- BDI
- ADS (german scale of CES-D)
- SCL90-D
- Depression index (DEPS)
- Zung
- Other

133. dep_pre_n

if seperate N is described for depression, please specify

134. dep_pre_m

135. dep_pre_sd

136. dep_post_n

137. dep_post_m

138. dep_post_sd

139. dep_fu1_t

dep: Follow up 1: specify months after end of treatment

140. dep_fu1_n

141. dep_fu1_m

142. dep_fu1_sd

143. dep_measurement_after_fu1

dep: Are there more follow-up measurements?

Mark only one oval.

- Yes *Skip to question 144*
- No *Skip to question 154*

depression_fu2

144. dep_fu2_t

dep: Follow up 2: specify months after end of treatment

145. dep_fu2_n

146. dep_fu2_m

147. dep_fu2_sd

148. dep_measurement_after_fu2

dep: Are there more follow-up measurements?

Mark only one oval.

- Yes
- No *Skip to question 154*

depression_fu3

this is the maximum amount of follow-up moments. Please list the final measurement.

149. dep_fu3_t

150. dep_fu3_n

151. dep_fu3_m

152. dep_fu3_sd

____

153. dep_measurement_after_fu3

dep: Are there any more follow-up measurements that you were not able to include?

Mark only one oval.

- Yes
- No

anxiety

Note: For questions about reporting the N -> if sepatate N is described for anx, please specify. Only fill in if the N deviates from the standard N, otherwise leave blank (fill in N if the author has applied imputation due to missing)

154. measurements_anxiety

Does the study include are measure of Anxiety?

Mark only one oval.

- Yes
- No *Skip to question 177*

anxiety_II

155. anx_name_measurement_instrument

anx: What is the name of the measurement instrument?

Mark only one oval.

- HADS-A
- SCL90-A
- STAI
- DASS: anxiety
- Other

156. anx_pre_n

if seperate N is described for Anxiety, please specify

157. anx_pre_m

158. anx_pre_sd

159. anx_post_n

160. anx_post_m

161. anx_post_sd

162. anx_fu1_t

anx: Follow up 1: specify months after end of treatment

163. anx_fu1_n

164. anx_fu1_m

165. anx_fu1_sd

166. anx_measurement_after_fu1

Mark only one oval.

- Yes
- No *Skip to question 177*

anxiety_fu2

167. anx_fu2_t

anx: Follow up 2: specify months after end of treatment

168. anx_fu2_n

169. anx_fu2_m

170. anx_fu2_sd

171. anx_measurement_after_fu2

Mark only one oval.

- Yes
- No *Skip to question 177*

anxiety_fu3

this is the maximum amount of follow-up moments. Please list the final measurement.

172. anx_fu3_t

anx: Follow up 3: specify months after end of treatment

173. anx_fu3_n

174. anx_fu3_m

175. anx_fu3_sd

176. anx_measurement_after_fu3

anx: Are there any more follow-up measurements that you were not able to include?

Mark only one oval.

- Yes
- No

general emotional functioning

Note: For questions about reporting the N -> if sepatate N is described for ef, please specify. Only fill in if the N deviates from the standard N, otherwise leave blank (fill in N if the author has applied imputation due to missing)

177. measurements_general_emotional_functioning

Does the study include are measure of General emotional functioning?

Mark only one oval.

- ( ) Yes
- ( ) No *Skip to question 200*

general_emotional_functioning_II

178. ef_name_measurement_instrument

Mark only one oval.

- ( ) DPQ: anxiety/depression
- ( ) NHP: Emotional reactions
- ( ) SF-36: mental health
- ( ) MPI: distress (0-12)
- ( ) Other

179. ef_pre_n

if seperate N is described for GEF, please specify

____

180. ef_pre_m

____

181. ef_pre_sd

____

182. ef_post_n

____

183. ef_post_m

184. ef_post_sd

185. ef_fu1_t
ef: Follow up 1: specify months after end of treatment

186. ef_fu1_n

187. ef_fu1_m

188. ef_fu1_sd

189. ef_measurement_after_fu1

Mark only one oval.

- ( ) Yes
- ( ) No *Skip to question 200*

general_emotional_functioning_fu2

190. ef_fu2_t

191. ef_fu2_n

192. ef_fu2_m

193. ef_fu2_sd

194. ef_measurement_after_fu2

Mark only one oval.

- Yes
- No *Skip to question 200*

general_emotional_functioning_fu3

this is the maximum amount of follow-up moments. Please list the final measurement.

195. ef_fu3_t

196. ef_fu3_n

197. ef_fu3_m

198. ef_fu3_sd

199. ef_measurement_after_fu3

Mark only one oval.

- Yes
- No

anger

Note: For questions about reporting the N -> if sepatate N is described for ang, please specify. Only fill in if the N deviates from the standard N, otherwise leave blank (fill in N if the author has applied imputation due to missing)

200. measurements_anger

Does the study include are measure of anger?

Mark only one oval.

- Yes
- No *Skip to question 223*

anger_II

201. ang_name_measurement_instrument

202. ang_pre_n

if seperate N is described for Anger, please specify

203. ang_pre_m

204. ang_pre_sd

205. ang_post_n

206. ang_post_m

207. ang_post_sd

208. ang_fu1_t

209. ang_fu1_n

210. ang_fu1_m

211. ang_fu1_sd

212. ang_measurement_after_fu1

Mark only one oval.

- Yes
- No *Skip to question 223*

anger_fu2

213. ang_fu2_t

____

214. ang_fu2_n

____

215. ang_fu2_m

____

216. ang_fu2_sd

____

217. ang_measurement_after_fu2

Mark only one oval.

- Yes
- No *Skip to question 223*

anger_fu3

this is the maximum amount of follow-up moments. Please list the final measurement.

218. ang_fu3_t

219. ang_fu3_n

220. ang_fu3_m

221. ang_fu3_sd

222. ang_measurement_after_fu3

ang: Are there any more follow-up measurements that you were not able to include?

Mark only one oval.

- Yes
- No

self_efficacy

Note: For questions about reporting the N -> if sepatate N is described for se, please specify. Only fill in if the N deviates from the standard N, otherwise leave blank (fill in N if the author has applied imputation due to missing)

223. measurements_self_efficacy

Does the study include are measure of self-efficacy?

Mark only one oval.

- Yes
- No *Skip to question 246*

self_efficacy_II

224. se_name_measurement_instrument

se: What is the name of the measurement instrument?

Mark only one oval.

- Dutch General Self-efficacy scale
- PSEQ
- PCL (negative SE)
- Other

225. se_pre_n

if seperate N is described for SE, please specify

226. se_pre_m

227. se_pre_sd

228. se_post_n

229. se_post_m

230. se_post_sd

231. se_fu1_t

232. se_fu1_n

233. se_fu1_m

234. se_fu1_sd

235. se_measurement_after_fu1

se: Are there more follow-up measurements?

Mark only one oval.

- Yes
- No *Skip to question 246*

self_efficacy_fu2

236. se_fu2_t

se: Follow up 2: specify months after end of treatment

237. se_fu2_n

238. se_fu2_m

239. se_fu2_sd

240. se_measurement_after_fu2

Mark only one oval.

- Yes
- No *Skip to question 246*

self_efficacy_fu3

this is the maximum amount of follow-up moments. Please list the final measurement.

241. se_fu3_t

242. se_fu3_n

243. se_fu3_m

244. se_fu3_sd

245. se_measurement_after_fu3

se: Are there any more follow-up measurements that you were not able to include?

Mark only one oval.

- Yes
- No

social_role_functioning

Note: For questions about reporting the N -> if sepatate N is described for srf, please specify. Only fill in if the N deviates from the standard N, otherwise leave blank (fill in N if the author has applied imputation due to missing)

246. measurements_social_role_functioning

Does the study include are measure of social role functioning?

Mark only one oval.

- Yes
- No *Skip to question 269*

social_role_functioning_II

247. srf_name_measurement_instrument

Mark only one oval.

- DPQ: social life
- Return to work
- Number of sick leave days
- SF-36: social functioning
- Other

248. srf_pre_n

if seperate N is described for SRF, please specify

____

249. srf_pre_m

250. srf_pre_sd

251. srf_post_n

252. srf_post_m

253. srf_post_sd

254. srf_fu1_t
srf: Follow up 1: specify months after end of treatment

255. srf_fu1_n

256. srf_fu1_m

257. srf_fu1_sd

258. srf_measurement_after_fu1

Mark only one oval.

- Yes
- No *Skip to question 269*

social_role_functioning_fu2

259. srf_fu2_t

260. srf_fu2_n

261. srf_fu2_m

262. srf_fu2_sd

263. srf_measurement_after_fu2

Mark only one oval.

- Yes
- No *Skip to question 269*

social_role_functioning_fu3

this is the maximum amount of follow-up moments. Please list the final measurement.

264. srf_fu3_t

265. srf_fu3_n

266. srf_fu3_m

267. srf_fu3_sd

268. srf_measurement_after_fu3

Mark only one oval.

- Yes
- No

pain_intensity

Note: For questions about reporting the N -> if sepatate N is described for pintens, please specify. Only fill in if the N deviates from the standard N, otherwise leave blank (fill in N if the author has applied imputation due to missing)

269. measurements_pain_intensity

Does the study include are measure of pain intensity?

Mark only one oval.

- Yes
- No *Skip to question 292*

pain_intensity_II

270. pintens_name_measurement_instrument

Mark only one oval.

- VAS (0-100)
- VAS (0-10)
- NRS (0-100)
- NRS
- Likert pain intensity
- NRS (0-10)
- PRI
- MPI: pain severity (0-6)
- MPI: pain severity (0-12)
- Other
- COMI: pain (0-10)

271. pintens_pre_n

if seperate N is described for pain intensity, please specify

272. pintens_pre_m

273. pintens_pre_sd

274. pintens_post_n

275. pintens_post_m

276. pintens_post_sd

277. pintens_fu1_t

278. pintens_fu1_n

279. pintens_fu1_m

280. pintens_fu1_sd

281. pintens_measurement_after_fu1

Mark only one oval.

- Yes *Skip to question 282*
- No *Skip to question 292*

pain_intensity_fu2

282. pintens_fu2_t

283. pintens_fu2_n

284. pintens_fu2_m

285. pintens_fu2_sd

286. pintens_measurement_after_fu2

Mark only one oval.

- Yes
- No *Skip to question 292*

pain_intensity_fu3

this is the maximum amount of follow-up moments. Please list the final measurement.

287. pintens_fu3_t

288. pintens_fu3_n

289. pintens_fu3_m

290. pintens_fu3_sd

291. pintens_measurement_after_fu3

Mark only one oval.

- Yes
- No

End of form

292. final follow-up

Please indicate the months of the final follow-up measurement

293. Do you have any comments or notes regarding the extraction?

This content is neither created nor endorsed by Google.

Google Forms
