# Supplementary material for: Longitudinal outcome evaluations of Interdisciplinary Multimodal Pain Treatment programmes for patients with chronic primary musculoskeletal pain: A systematic review and meta‐analysis
Source: Eur J Pain. 2021 Nov 5;26(2):310–35. doi: 10.1002/ejp.1875 (PMC9297911; doi:10.1002/ejp.1875)
Supplement: Supplementary file 3 — Supplementary Material [file EJP-26-310-s002.docx]

**Appendix 1. Risk of Bias Assessment**

| **Author (year)** | **1** | **2** | **3** | **4** | **5** | **6** | **7** | **8** | **9** | **10** | **Decision** |
| --- | --- | --- | --- | --- | --- | --- | --- | --- | --- | --- | --- |
| Abbasi  (2012) | + | + | + | + | - | - | + | - | + | ? | high |
| Beaudreuil (2010) | + | + | + | - | - | + | + | + | + | ? | high |
| Bendix  (1998) | + | + | + | ? | + | + | - | + | + | - | high |
| Bendix (2000) | - | - | + | ? | + | + | - | + | + | - | high |
| Bergström (2001) | - | + | + | + | + | + | + | + | + | ? | high |
| Bergström (2014) | + | + | + | - | + | + | - | + | + | - | high |
| Bileviciute-Ljungar (2014) | - | + | + | + | - | + | + | + | + | - | high |
| Borys  (2015) | + | + | + | + | - | - | + | + | + | - | high |
| Brendbekken (2016) | - | - | + | ? | - | + | - | + | + | - | high |
| Cardosa (2012) | - | + | + | ? | + | + | + | + | + | - | high |
| De Rooij (2014) | + | + | + | + | - | + | + | + | + | + | high |
| Dysvik (2013) | + | + | + | + | + | + | + | + | + | - | high |
| Frost  (2000) | + | + | + | - | ? | - | - | + | + | - | high |
| Gantschnig (2017) | - | - | + | + | + | + | - | + | + | + | high |
| Gerdle  (2016) | - | - | + | + | ? | + | + | + | + | - | high |
| Grahn  (2000) | + | - | + | + | + | + | + | + | + | - | low |
| Gustafsson (2002) | + | + | + | - | + | + | + | + | + | - | high |
| Hafenbrack (2013) | - | - | + | + | + | + | - | + | + | - | high |
| Haiduk (2017) | + | + | + | + | + | + | ? | + | + | ? | high |
| Hållstam (2016) | - | + | + | + | ? | + | + | + | + | - | high |
| Hazard (1989) | + | + | + | + | - | + | - | + | + | - | high |
| Hildebrandt (1996) | - | + | + | ? | ? | + | - | + | + | - | high |
| Huffman (2019) | - | - | + | + | + | + | - | + | + | - | high |
| Ibrahim (2019) | - | - | + | + | ? | + | - | + | + | - | high |
| Jensen  (1997) | - | + | + | - | - | + | + | + | + | + | high |
| Kääpä  (2006) | + | + | + | - | + | - | + | + | + | ? | high |
| Koopman (2004) | + | + | + | ? | ? | + | + | + | + | - | high |
| Lemstra (2005) | + | + | + | + | + | + | + | + | + | - | low |
| Letzel  (2019) | + | + | + | + | ? | + | - | + | + | - | high |
| Mangels (2009) | - | - | + | - | - | + | - | + | + | ? | high |
| Martín  (2012) | + | + | + | + | + | + | + | - | + | - | high |
| McAllister (2005) | + | + | + | + | ? | + | - | + | - | - | high |
| Meng  (2011) | - | - | + | ? | - | + | - | + | + | - | high |
| Merrick (2009) | + | + | + | + | + | + | + | + | + | - | high |
| Merrick (2012) | + | - | + | + | + | + | + | + | + | + | high |
| Monticone (2013) | + | + | + | + | - | + | + | + | + | + | high |
| Monticone (2016) | + | + | + | + | - | - | + | + | + | - | high |
| Nagel  (2009) | - | + | + | + | ? | - | + | + | + | - | high |
| Nicholas (2020) | + | + | + | + | + | + | + | + | + | - | high |
| Olason  (2004) | - | + | + | - | - | + | + | + | + | - | high |
| Oslund (2009) | - | + | - | - | ? | + | + | + | + | ? | high |
| Persson (2012) | - | + | + | ? | ? | + | + | + | + | - | high |
| Pietilä-Holmner (2020) | - | ? | + | + | + | + | + | + | + | - | high |
| Reck  (2017) | - | + | + | + | + | + | + | + | + | - | high |
| Richardson (1994) | - | - | + | ? | ? | + | - | + | + | - | high |
| Roche-Leboucher (2011) | + | + | - | + | ? | - | - | + | + | - | high |
| Semrau (2015) | + | + | + | + | + | + | + | + | + | + | low |
| Silvemark (2014) | - | + | + | + | + | + | + | + | + | - | high |
| Smeets (2008) | + | + | + | + | - | + | + | + | + | + | high |
| Spinhoven (2004) | + | + | + | - | - | + | + | + | + | ? | high |
| Stein  (2013) | + | + | + | - | - | + | + | - | + | - | high |
| Steinmetz (2019) | - | - | + | ? | ? | - | - | + | + | - | high |
| Strobel (1998) | - | + | + | ? | ? | + | + | - | + | - | high |
| Tavafian (2011) | + | + | + | + | + | + | + | + | + | - | low |
| Thieme (2003) | + | + | + | - | - | + | + | + | + | - | high |
| Van der Maas (2015) | - | + | + | + | - | + | + | + | + | + | high |
| van Hooff (2010) | + | + | + | + | - | + | + | + | + | + | high |
| van Wilgen (2009) | + | + | + | - | ? | + | + | + | + | - | high |
| Vendrig (2000) | - | + | + | + | + | + | + | + | + | - | high |
| Verkerk (2011) | + | + | + | - | - | + | + | + | + | ? | high |
| Volker (2017) | + | + | + | + | + | + | + | + | + | + | low |
| Vowles  (2011) | + | + | + | + | + | + | + | + | + | + | low |
| Wagner (2011) | - | - | + | + | - | + | - | + | + | ? | high |
| Westman (2006) | - | - | + | + | ? | + | - | - | + | - | high |
| Williams (1996) | + | + | + | + | - | + | - | - | + | - | high |
| Zhuk  (2018) | + | + | + | ? | ? | + | - | + | + | - | high |
